# Inhibition of Histone Demethylases LSD1 and UTX Regulates ERα Signaling in Breast Cancer

**DOI:** 10.3390/cancers11122027

**Published:** 2019-12-16

**Authors:** Rosaria Benedetti, Carmela Dell’Aversana, Tommaso De Marchi, Dante Rotili, Ning Qing Liu, Boris Novakovic, Serena Boccella, Salvatore Di Maro, Sandro Cosconati, Alfonso Baldi, Emma Niméus, Johan Schultz, Urban Höglund, Sabatino Maione, Chiara Papulino, Ugo Chianese, Francesco Iovino, Antonio Federico, Antonello Mai, Hendrik G. Stunnenberg, Angela Nebbioso, Lucia Altucci

**Affiliations:** 1 Department of Precision Medicine, University of Campania “Luigi Vanvitelli”, 80138 Naples, Italy; carmela.dellaversana@unicampania.it (C.D.); chiara.papulino@unicampania.it (C.P.); ugo.chianese@unicampania.it (U.C.); angela.nebbioso@unicampania.it (A.N.); 2 Institute Experimental Endocrinology and Oncology “Gaetano Salvatore” (IEOS)-National Research Council (CNR) Via Sergio Pansini, 5-80131 Napoli, Italy; 3 Department of Oncology and Pathology, Lund University, SE-221 00 Lund, Sweden; tommaso.de_marchi@med.lu.se (T.D.M.); emma.nimeus@med.lu.se (E.N.); 4 Department of Drug Chemistry and Technologies, Sapienza University of Rome, 00185 Rome, Italy; dante.rotili@uniroma1.it (D.R.); antonello.mai@uniroma1.it (A.M.); 5 Department of Molecular Biology, Radboud University, 6500 HB Nijmegen, The Netherlands; n.liu@nki.nl (N.Q.L.); H.Stunnenberg@ncmls.ru.nl (H.G.S.); 6 Murdoch Children’s Research Institute and Department of Paediatrics, University of Melbourne, Melbourne, Parkville Victoria 3052, Australia; boris.novakovic@mcri.edu.au; 7 Department of Experimental Medicine, Section of Pharmacology “L. Donatelli”, University of Campania “Luigi Vanvitelli”, 80138 Naples, Italy; boccellaserena@gmail.com (S.B.); sabatino.maione@unicampania.it (S.M.); 8 Dipartimento di Scienze e Tecnologie Ambientali Biologiche e Farmaceutiche, University of Campania ’Luigi Vanvitelli’, 81100 Caserta, Italy; salvatore.dimaro@unicampania.it (S.D.M.); sandro.cosconati@unicampania.it (S.C.); alfonsobaldi@tiscali.it (A.B.); 9 Department of Surgery, Skånes University Hospital, 222 29 Lund, Sweden; 10 Kancera AB, Banvaktsvagen 22, SE-17148 Solna, Sweden; johan.schultz@kancera.com; 11 Adlego Biomedical AB, P.O. Box 42, SE-751 03 Uppsala, Sweden; Urban.Hoglund@adlego.se; 12 Department of Translational Medical Sciences, University of Campania “Luigi Vanvitelli”, Via L. De Crecchio 7, 80138 Naples, Italy; francesco.iovino@unicampania.it; 13 Faculty of Medicine and Health Technology, Tampere University, 33100 Tampere, Finland; antoniofedigb@gmail.com; 14 Prinses Maxima Centrum, Heidelberglaan 25, 3584CS Utrecht, The Netherlands

**Keywords:** KDM inhibitor 1, LSD1 2, UTX 3, ERα 4, hormone signaling 5

## Abstract

In breast cancer, Lysine-specific demethylase-1 (LSD1) and other lysine demethylases (KDMs), such as Lysine-specific demethylase 6A also known as Ubiquitously transcribed tetratricopeptide repeat, X chromosome (UTX), are co-expressed and co-localize with estrogen receptors (ERs), suggesting the potential use of hybrid (epi)molecules to target histone methylation and therefore regulate/redirect hormone receptor signaling. Here, we report on the biological activity of a dual-KDM inhibitor (MC3324), obtained by coupling the chemical properties of tranylcypromine, a known LSD1 inhibitor, with the 2OG competitive moiety developed for JmjC inhibition. MC3324 displays unique features not exhibited by the single moieties and well-characterized mono-pharmacological inhibitors. Inhibiting LSD1 and UTX, MC3324 induces significant growth arrest and apoptosis in hormone-responsive breast cancer model accompanied by a robust increase in H3K4me2 and H3K27me3. MC3324 down-regulates ERα in breast cancer at both transcriptional and non-transcriptional levels, mimicking the action of a selective endocrine receptor disruptor. MC3324 alters the histone methylation of ERα-regulated promoters, thereby affecting the transcription of genes involved in cell surveillance, hormone response, and death. MC3324 reduces cell proliferation in ex vivo breast cancers, as well as in breast models with acquired resistance to endocrine therapies. Similarly, MC3324 displays tumor-selective potential in vivo, in both xenograft mice and chicken embryo models, with no toxicity and good oral efficacy. This epigenetic multi-target approach is effective and may overcome potential mechanism(s) of resistance in breast cancer.

## 1. Introduction

Breast cancer (BC) is the most frequent cancer in women (American Institute for Cancer Research) [1]. Most BCs are estrogen receptor (ER)α positive, and both clinical observations and laboratory studies suggest that ERα signaling pathway is the major driver in promoting proliferation, survival, and invasion [2,3,4]. Endocrine therapy is the mainstay of treatment for patients with ERα-positive BC [4]. In hormone-sensitive BC, tamoxifen acts as a partial antagonist and belongs to the class of selective estrogen receptor modulators (SERMs). However, tamoxifen treatment frequently leads to resistance, making therapy ineffective in the long term (10–15% of patients with early-stage ERα-positive BC within 5 years) [5,6]. Interestingly, many patients who relapse on tamoxifen therapy will respond to different ERα downregulators (e.g., fulvestrant), acting as selective endocrine receptor disruptor (SERD) [7]. The majority of tamoxifen-resistant ERα-positive BC is still sensitive to fulvestrant, although it requires intramuscular injection, and a complex dosing schedule, limiting its application in a neoadjuvant setting [8,9,10]. Current research for SERD molecules in BC seems more promising, due to their intrinsic property of inducing only limited phenomena of resistance. However, in different phases of BC progression ERα signaling is mediated by genomic and non-genomic estrogen actions, both contributing to cell migration, motility, and survival. A complex epigenetic regulation underlies the function of ERα as a transcription factor, leading to the hypothesis that the inhibition of epigenetic enzymes could be an advantageous strategy for BC treatment. In human BC, ERα seems to functionally associate with several lysine (K)-specific demethylases (KDMs), such as LSD1, able to modulate its transcriptional activity [11,12,13,14]. The same holds true for UTX (KDM6A), an H3K27 demethylase mainly associated with gene activation [15,16,17]. The function of both enzymes was recently shown to be crucial for ERα transcriptional activity [17]. These findings provide the rationale for using in BC a dual epigenetic KDM inhibitor directed against LSD1 and UTX to reduce breast cancer cell proliferation, invasiveness, and metastatic capability. Here, we describe and characterize a novel dual-KDM inhibitor (MC3324) [18], obtained by coupling the chemical properties of tranylcypromine (TCP), a known LSD1 inhibitor, with the 2OG competitive moiety developed for Jumonji C domain-containing protein (JmjC)-KDM inhibition [19]. MC3324 displays unique features not exhibited by single scaffolds (TCP and 2OG) and well-characterized specific LSD1 and UTX inhibitors. In BC cells, MC3324 mimics the activity of a SERD, reducing ERα at transcriptional and protein level. Downregulation of ERα is accompanied by epigenetic regulation of ERα and ERα-responsive promoters, with a global and region-specific increase in H3K4me2 and H3K27me3 after few hours of treatment. This effect creates a bridge between epigenetic regulation occurring via multiple KDM inhibition and ERα signaling cascade, leading to activation/repression of biological pathways that generate an immediate readout on cell proliferation, migration, and death.

## 2. Results

### 2.1. MC3324 Is a Dual LSD1 and UTX Inhibitor Regulating ERα Signaling

**Figure 1:**
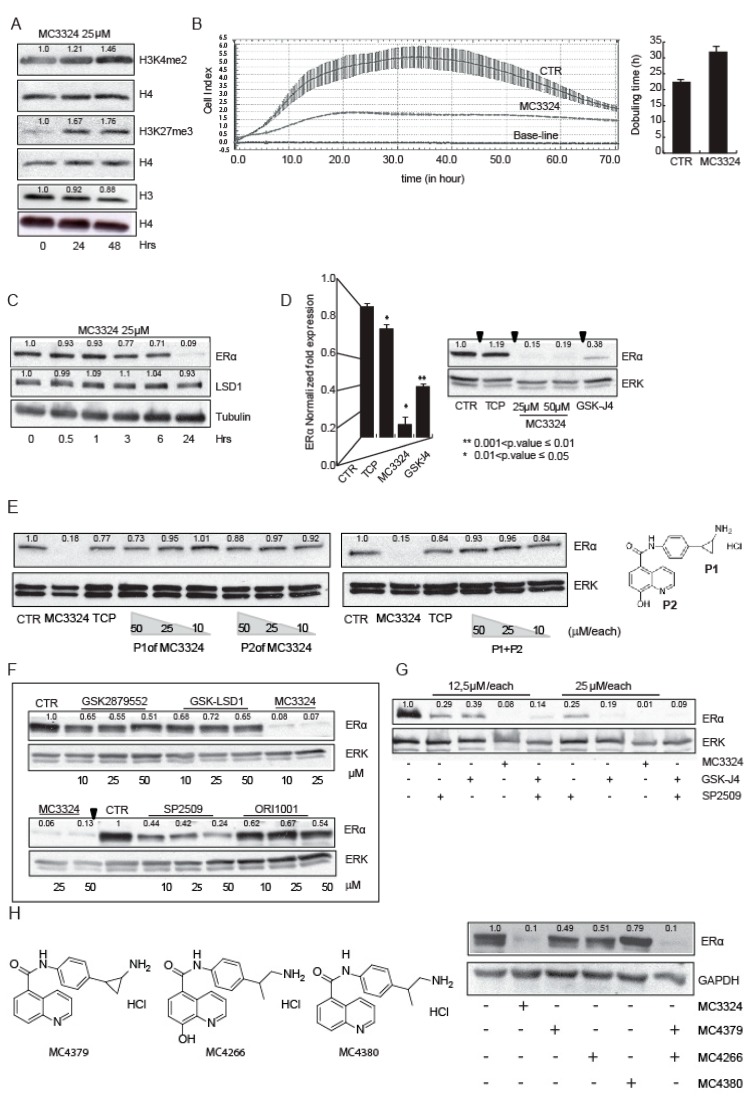
MC3324 is a LSD1/UTX inhibitor and regulates estrogen receptor (ER)α expression and cell proliferation in MCF7 cell line. (**A**) Histone methylation levels after MC3324 treatment (25 µM) in MCF7. The increase in dimethylation of histone H3 at lysine K4 and trimethylation of K27, respectively were evaluated after 24 h and 48 h post induction. The relative increase was quantified with ImageJ software (1.46r, NIH, USA). The level of H3 is almost unchanged with MC3324 treatment. (**B**) Proliferation arrest induced with MC3324 at the dose of 25 µM. Cell Index was measured in real-time up to 70 h. The experiment was performed in triplicate. (**C**) Time course of ERα and LSD1 expression levels after the induction with MC3324 in MCF7. (**D**) mRNA evaluation and protein expression of ERα after induction with MC3324 (25 µM and 50 µM), tranylcypromine (TCP) (100 µM) and GSK-J4 (25 µM) for 24 h. (**E**) ERα expression after induction with MC3324 scaffolds, alone and in combination at indicated doses. (**F**) ERα modulation with commercial LSD1 inhibitors at indicated doses. (**G**) ERα modulation with commercial LSD1 and UTX inhibitors, alone and in combination. (**H**) ERα expression modulated by MC3324 derivatives (25 µM), lacking one or both inhibitory activities.

In MCF7 cells, MC3324 inhibited LSD1 and UTX and induced a time-dependent increase in dimethylation of histone H3 at lysine K4 and trimethylation of K27, respectively (Figure 1A). This effect was coupled with the proliferation arrest and with the increase of cellular doubling time (Figure 1B). Cellular thermal shift assay (CETSA) confirmed the binding and the physical interaction of MC3324 with LSD1 and UTX (Figure S1A), which were both protected from thermal degradation. Theoretical studies provided a clearer picture, at molecular level, of binding interactions between MC3324 and UTX. Specifically, the ligand is able to chelate the Fe^2+^ ion within the binding cavity through its 8-hydroxyquinoline moiety. Moreover, decoration of the compound with TCP portion, as LSD1 inhibitor, allows the ligand to form additional H-bond interactions with the enzyme counterpart, thereby suggesting a tight binding of MC3324 with UTX. In BC, MC3324 induced time/dose-dependent downregulation of ERα at protein and mRNA level respectively (Figure 1C,D). Compared to TCP and GSK-J4 (commercially available inhibitors of LSD1 and UTX, respectively) MC3324 showed a stronger ERα downregulation (Figure 1D). Interestingly, the two moieties of MC3324 (reported in Figure 1E as P1 and P2), alone or in combination in escalating doses, did not induce ERα downregulation (Figure 1E). MC3324 was also compared to other KDM inhibitors alone and in co-treatment (Figure 1F,G). GSK2879552 reduces by approximately 50% the expression of ERα, GSK-LSD1 and ORI1001 used as a single inhibitor are only weakly effective and exclusively at high doses (Figure 1F). SP2509 alone induces about 60% of ERα downregulation, therefore it was tested together with the UTX inhibitor. Only the combination of GSK-J4 and SP2509, had a similar effect to that of MC3324 (Figure 1G), although downregulation of the ERα was obtained with a double dose of each inhibitor. Modifying the chemical structure of MC3324 by alternatively abrogating the LSD1 and UTX inhibition potential (in Figure 1H MC4379, MC4266, MC4380, and see supplementary materials for Structure-Activity Relation Study, SAR study) attenuated downregulation of ERα. This mini SAR Study strongly corroborated the idea that simultaneous inhibition of LSD1 and UTX is required to silence ERα and its signaling and that the use of a single (double acting) agent had a more potent effect compared to the combination of two drugs, at the same dose. In MCF7 cells, MC3324 induced a block in G1 phase accompanied by induction of pre-G1 accumulation and a reduction in cell migration (Figure S1B). Following inhibition, LSD1 protein levels did not vary (Figure 1C), while UTX was downregulated (Figure S1C). Although the hypothesized mechanism of action of MC3324 is non-covalent, reversible inhibitor, this can be justified by the different affinity of MC3324 to LSD1 and UTX. Supporting MC3324 anticancer activity, the LSD1 and UTX inhibition impacted on expression of proteins involved in cell death and cell cycle (Figure S1C), inducing apoptosis and a block in proliferation. The importance of LSD1 for ERα protein expression and signaling was confirmed by the observed downregulation of ERα when LSD1 was decreased (Figure S1D). Moreover, decreased expression of LSD1 reduced the proliferative index of MCF7 cells, confirming LSD1 activity as a tumor promoter (Figure S1E).

**Figure 2:**
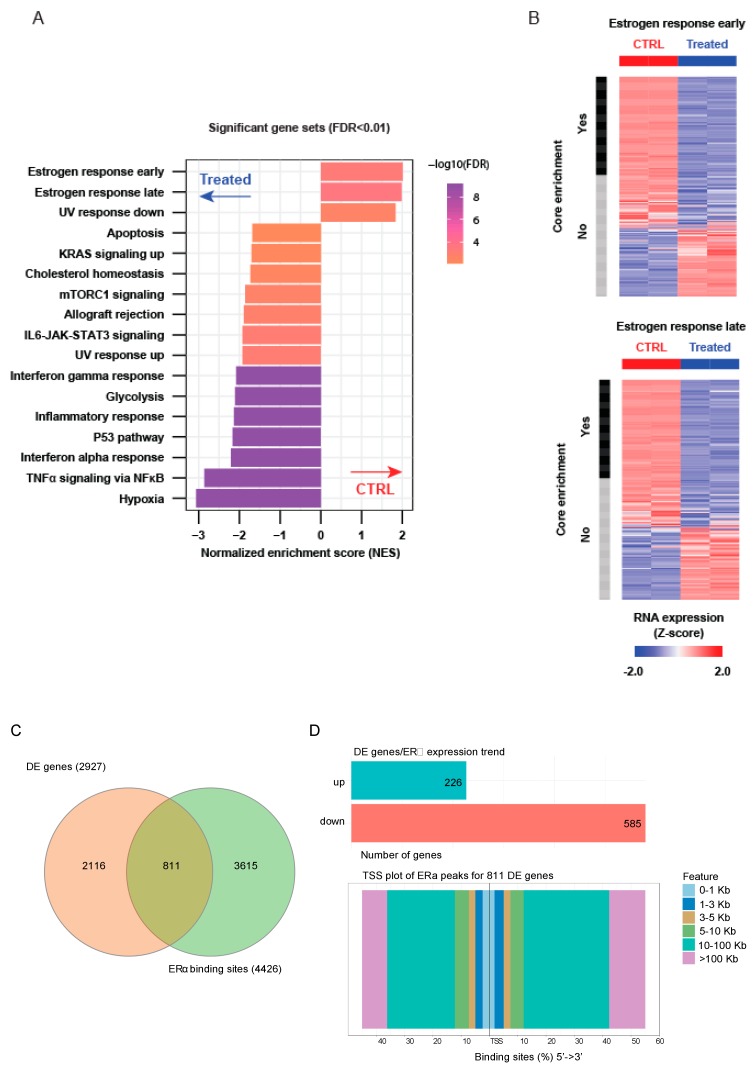
MC3324 regulates transcription and ERα signaling in MCF7 cells. (**A**) Gene set enrichment analysis (GSEA) of MC3324 regulated genes after 24 h of treatment in MCF7. (**B**) Expression of 2 most enriched gene sets in MCF7 untreated. (**C**) Venn diagram summarizing results relative to deregulated mRNA co-associated with ERα binding sites. (**D**) Barplot of up/down-regulated genes associated with ERα binding sites. TSS plot of 811 regulated genes is reported.

RNA-seq profiling following LSD1 and UTX inhibition for 24 h revealed the activation of pathways related to cell death and cell cycle modulation in MC3324-treated MCF7 cells (Figure 2A), probably involving activation of p53 pathway (also increased at protein level; Figure S1C). The hallmarks of estrogen response were downregulated in MC3324-treated MCF7 cells (Figure 2A,B). Table S1 reports the list of differentially expressed genes after MC3324 induction and Table S2 shows top up/down-ranked pathways. Figure S2 shows gene plots reported in the main Figure 2. Comparison of 2933 differential expression genes (Table S1) with public data sets for ERα binding sites (Table S3) identified 811 genes as ERα targets (Figure 2C,D), revealing that ERα downregulation obtained with MC3324 strongly impacts on expression of key genes. Genes with ERα binding sites are mostly downregulated (Figure 2D) and moreover, to restrict the analysis to the ERα binding sites surrounding the Transcription Start Site (TSS) of Differentially expressed (DE) genes, ERα peaks within the range of 0–1kb were considered. Specifically, upstream the TSS 33 out of 48 DE genes were downregulated (corresponding to 68.7%), while downstream 23 out of 40 DE genes were downregulated (corresponding to 57.5%). These results confirm that downregulated genes possess a higher amount of ERα binding sites surrounding their TSS. The same analysis was also done with public data set for LSD1 and UTX (GSE104755 and GSE96996, respectively) and results are reported in Figure S3 and Table S4 and Figure S4 and Table S5. These data suggest that LSD1 and UTX are required for proliferation of BC and that both enzymes are involved in the control of estrogen pathway in MCF-7 cells. 

### 2.2. MC3324 Blocks Proliferation of Tamoxifen-Insensitive BC Cell Line

**Figure 3:**
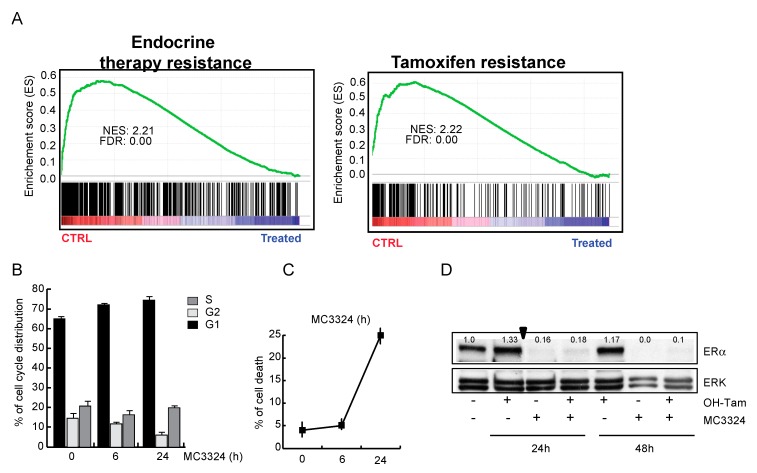

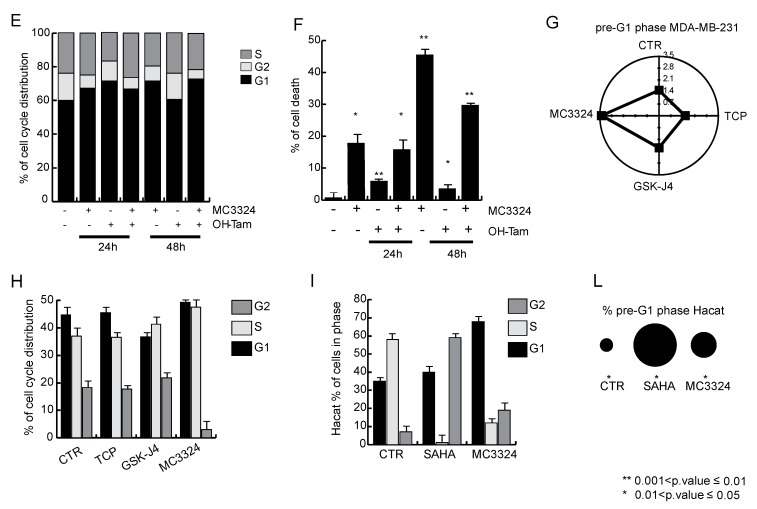
MC3324 activities in BT474, MDA-MB-231, and HaCaT cellular models. (**A**) Enrichment plot in MCF7 showing that MC3324 overcomes resistance mechanisms. BT474 cell cycle distribution (**B**) and cell death induction (**C**) after treatment with MC3324 (25 µM) for 24 h. Time dependent ERα down regulation in BT474 (**D**) following MC3324 treatment (25 µM) is associated with cell cycle arrest (**E**) and induction of cell death (**F**). In MDA-MB-231 cells, MC3324 does not induce cell death (**G**) and cell phase’s perturbation (**H**) after 24 h of induction at the concentration of 25 µM. In non-cancerous cells (HaCaT) MC3324 has weak pro-death effects (**I–L**) when used at 25 µM for 24 h. The calculated percentage of cell death is CTR: 5%, SAHA: 30% and MC3324: 12%.

Downregulation of ERα, accompanied by a reduction in the proliferative potential of MCF7, suggests that MC3324 is mimicking the action of a selective endocrine receptor disruptor (SERD), promoting dowregulation of genes involved in endocrine therapy response and tamoxifen resistance mechanisms (Figure 3A and Table S2). MC3324 anticancer effect is mediated by an epigenetic regulation of ERα activity, highlighting the existence of a druggable axis between LSD1/UTX inhibition and response to hormones. To exploit the possibility of using MC3324 in BC with innate or acquired resistance to endocrine therapy, tamoxifen-insensitive-ERα positive BT474 cells were treated for 24 and 48 h with MC3324 alone or in combination with tamoxifen (Figure 3B–F). In this cell line, the decrease in ERα levels was again coupled with a reduction in proliferative index and induction of cell death. In contrast, in MDA-MB-231 cells (Figure 3G–H), used as a model of triple-negative BC, MC3324 did not induce cell death, but only a weak cell cycle perturbation and S phase accumulation, underscoring the importance of ERα to achieve epigenetic rebalance, possibly via activation of the intracellular cascade in which ERα is, both, effector and target. To confirm the anti-cancer selective action of MC3324, a normal immortalized cell line (HaCaT) were treated. In this cell line, MC3324 displayed low toxicity compared to Suberoylanilide Hydroxamic Acid (SAHA), a well-known epigenetic drug acting as histone deacetylase inhibitor (Figure 3I,L). Interestingly, and as expected for the known overexpresson of LSD1 and UTX in cancer models, the expression levels of LSD1 and UTX are different in MCF7, MDA-MB-231 breast cancer vs HaCaT normal keratinocytes cells (Figure S1F), suggesting that also these features might contribute to the anticancer action identified. Together these data suggest that blocking ERα expression by UTX and LSD1 inhibition is a valid alternative to interfere with hormonal pathways and to induce cell death and growth arrest also in tamoxifen-insensitive cells. 

### 2.3. LSD1 and UTX Inhibition Modulates ERα Interactome and Hormone Signaling Cascade

**Figure 4:**
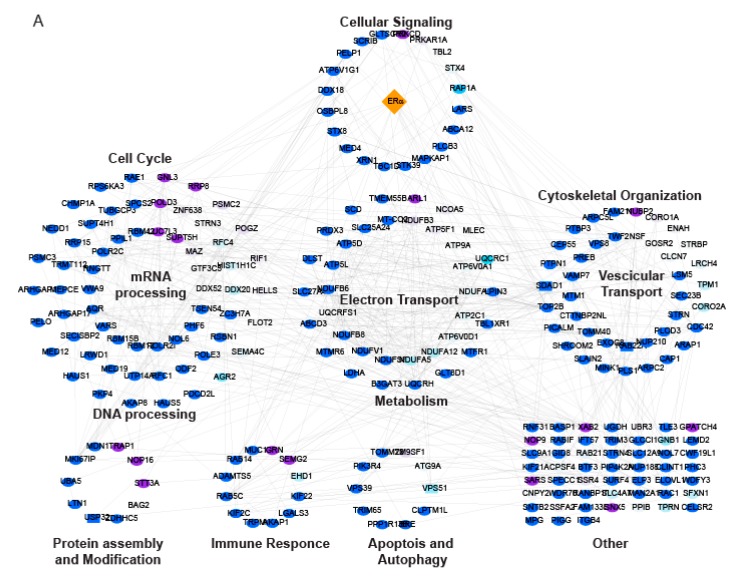

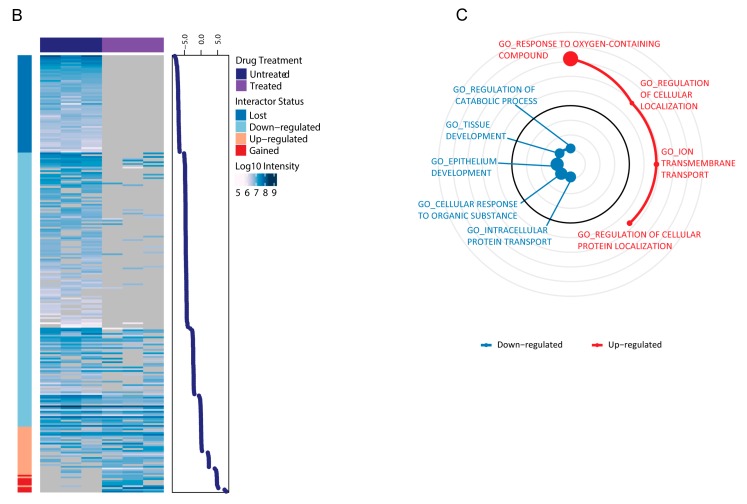
ERα interaction network changes following MC3324 treatment. (**A**) Proteins identified by ERα pulldown after the treatment with MC3324 (25 µM for 6 h) were annotated and clustered based on Gene Ontology Biological Process (GOBP) terms and visualized as a STRING (www.string-db.org) network in Cytoscape. Nodes represent identified proteins; edges represent interactions derived from the STRING database. Node color code: pulldown target (orange), upregulated interactor (purple), down-regulated interactor (light blue). Heatmap of ERα interactors (**B**) shows a great number of ERα interactors were either lost (no observation in treated) or down-regulated (negative Log2 Ratio) after MC3324 treatment, while only a handful of interactors were up-regulated (positive Log2 Ratio) or gained (no observation in untreated). GSEA was performed to assess which pathways (**C**) displayed significant regulation following MC3324 treatment.

The overlap between the effects of LSD1 and UTX inhibition on gene transcription with those prompted by ERα downregulation indicated that both enzymes may have a pivotal role in the control of the estrogen pathway in MCF-7 cells. For this reason, ERα interactome was analysed to assess potential deregulation of interactors binding following MC3324 treatment for 6 h. After MC3324 induction, modified ERα interactors were found and grouped by biological pathways, revealing a major shift in biological signaling at the protein level (Figure 4A–C and Table S6). The top enriched pathways by gene set enrichment analysis (GSEA) (FDR (false discovery rate) < 0.25) with (upper) and without (lower) treatment are shown in Figure 4C. In MC3324-treated conditions, upregulation of pathways related to response to oxygen/nitrogen-containing compounds was observed, as was a perturbation in regulation of cellular (protein) localization, while pathways associated with epithelium and tissue development were downregulated. Differential ERα interactors in MC3324-treated and untreated MCF7 cells also impacted on regulation of cellular response to steroid hormone stimulus and ERα stability via decreasing interaction with HNRPU, SAFB, RBM14, DDX54, ROCK2, MED12/4, EP300, and PELP1 and MUC1 [20,21,22] (Table S6). In our experimental conditions, we were not able to detect a direct physical interaction between ERα, LSD1, and UTX; this was confirmed by reverse immunoprecipitation against LSD1 (Figure S5A–C and Table S6). Few interactors were common to ERα and LSD1 pull-down experiments (represented by a rectangle in networks in Figure 4A and Figure S5A). As MC3324 directly binds LSD1 [18], it induced changes in LSD1 interactors leading to downregulation of protein phosphorylation, chromosome organization, and cell death pathways (Figure S5C, GSEA). The list of GSEA enriched pathways for ERα and LSD1 is reported in Table S6. As some altered ERα and LSD1 interactors were also differentially expressed (Figure S6A,B and Figure S7A,B), the epigenetic modulation obtained with MC3324 both regulated the cellular transcriptome in MCF7 cells and impacted on intracellular macrocomplexes. These two combined effects led to the down regulation of hormonal signaling and activation of cell death mechanisms. We concluded that MC3324 inhibits LSD1 and UTX activity, inducing epigenetic changes at ERα promoter, causing the decrease of ERα transcription and triggering a regulation mechanism whereby ERα downregulation prevents the receptor from acting as a transcriptional factor, changes its interactors and directs it towards degradation depending on its turnover. Inhibition of both enzymes induced a specific reprogramming of the hormone-responsive BC cell transcriptome, determining functional consequences in the ERα interactome, shifting the cells to the programmed cell death activation pathway.

### 2.4. Epigenetic Rebalance of Erα Signaling via LSD1 and UTX Inhibition

**Figure 5:**
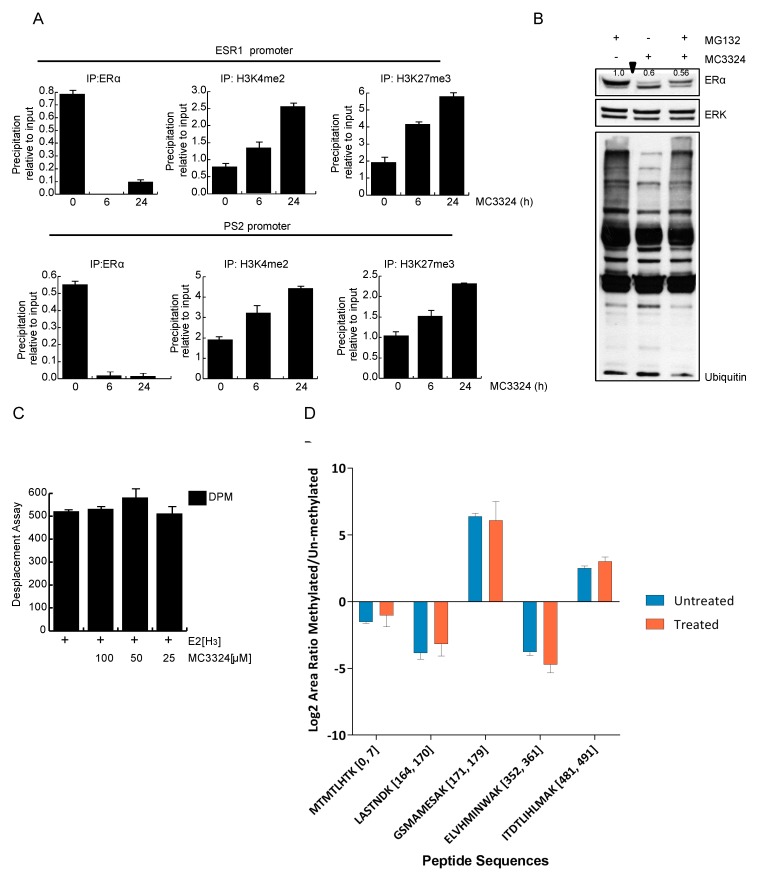
MC3324 increases H3K4me2 and H3K27me3 on ERα regulated promoters. (**A**) Chromatin immunoprecipitation (ChIP) experiments in MCF7 after MC3324 treatment on ERα and PS2 promoters. Data are normalized on IgG. (**B**) ERα down regulation is not restored after block (MG132 for 6 h at the concentration of 10 μM) of proteasomal degradation. (**C**) MC3324 does not bind ERα in radiolabeled in vitro assay. (**D**) MS of IP:ERα does not revel methylated lysines after MC3324 treatment for 6 h at (25 µM) in MCF7 cells. Results are the average of independent triplicates.

Chromatin immunoprecipitation (ChIP) experiments showed that after induction with MC3324 for 6 h and 24 h, H3K4me2 and H3K27me3 levels increased, while ERα occupancy on its own and on PS2 promoter decreased in a time-dependent manner (Figure 5A). At 6 h treatment, ERα was still present in cells, but no longer found on its promoter, suggesting that the increase in H3K4me2 and H3K27me3 blocks ERα binding to its promoter and prevents its transcription (Figure 5A). Epigenetic regulation of ERα was in line with the only partial rescue of receptor levels after block of proteasomal degradation, corroborating the idea that MC3324 affects ERα expression rather than protein stability (Figure 5B). Additionally, MC3324 did not act directly as a ligand of ERα, as shown by radiolabeled displacement assay (Figure 5C). ERα itself is not a direct substrate of LSD1 and/or UTX, and no methylation changes on several lysine residues (Figure 5D) were observed. After immunoprecipitation, ERα peptides originated by tryptic digestions were analyzed by MS/MS (Table S7). Although none of the detectable peptides seemed to be methylated after MC3324 treatment, this result is very preliminary and should be further investigated, perhaps using orthogonal methods. Together, these results point to the crucial role of LSD1 and UTX in controlling ERα expression and activity in BC and underscore the possibility of epigenetically rebalancing BC via the pharmacological intervention with MC3324.

### 2.5. MC3324 Displays Anticancer Action In Vivo in Both Chicken Embryo and Mouse Models and Ex Vivo in Human BC Specimens

**Figure 6:**
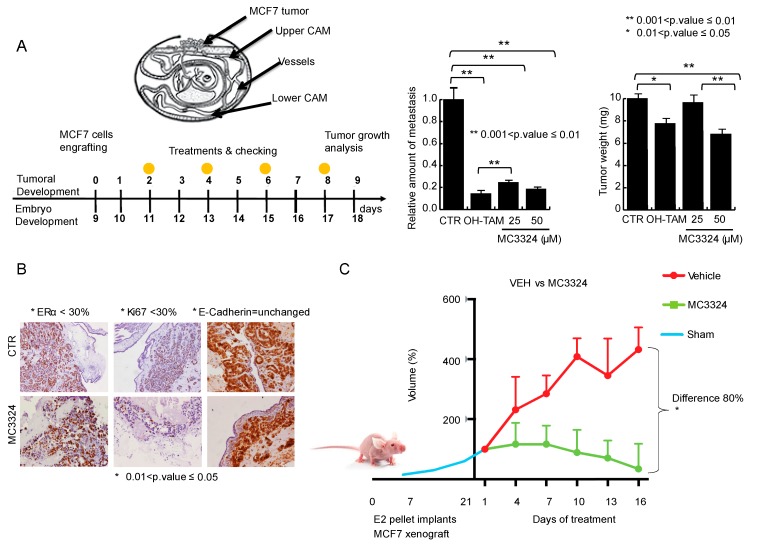

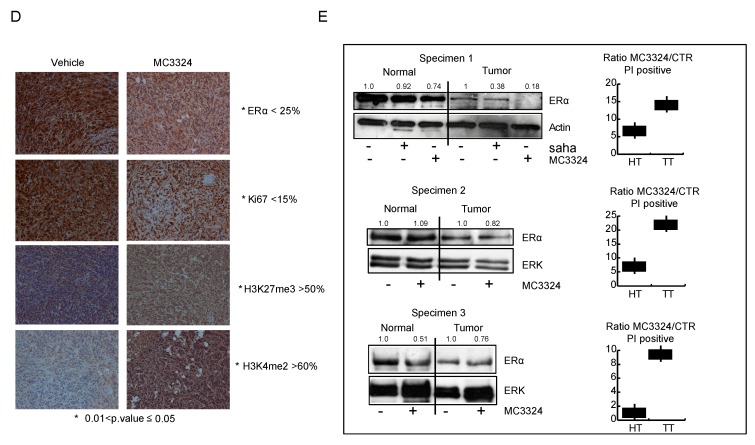
In-vivo and ex-vivo anticancer effects of MC3324. (**A**) General schematic of chicken embryos engrafted with MCF7 cells, anti-proliferative effect and reduction of migration. (**B**) Immunostaining of MCF7 cells after MC3324 treatment (time and concentrations reported in figure). (**C**) MCF7 xenograft model showing MC3324 anticancer effects. Successful tumor engraftment of MCF7 in nude mice was of 60%. Data are the average volumes of 6 independent mouse engrafted for MC3324 treated and vehicle. (**D**) Immunostaining on mice isolated tumors treated and untreated (vehicle) with MC3324. (**E**) MC3324 induces cell death in ex vivo breast specimens after 24 h treatment (HT = Healthy Tissue; TT = Tumor Tissue). Cell death evaluation in ex-vivo cells from healthy surrounding and tumor tissues was reported as Ration between propidium iodide (PI) positive cells after MC3324 treatment for 24 h. Cells were also blotted for ERα.

Experiments on chicken embryos represented a breakthrough in determining the potential application of MC3324 in anticancer therapy. In this model of tumor development and maintenance, MC3324 was able to reduce tumor size and completely abolish the migratory potential of BC cells compared to tamoxifen (Figure 6A). The percentage of tumor regression coupled with the absence of toxicity is reported in Figure S8A,B. The reduction in proliferative potential was confirmed by Ki-67 staining of MCF7-derived masses. The reduction in Ki-67 was accompanied by a reduction in ERα and no variation in E-cadherin (Figure 6B). In a mini pharmacokinetic study (Figure S8C,E), followed by xenograft experiments, MC3324 proved to be stable and nontoxic, well-tolerated, and effective when orally administered. Two mice oral administered (per os; p.o.) with MC3324 showed plasma concentrations of 15–40 μM at the 30 and 60 min time points. At 180 min post-administration, plasma concentrations were 5 μM and 7 μM. In xenograft experiments, the reduction in tumor size was measured as the differences in volume and lateral dimensions (Figure 6C) and staining experiments were performed (Figure 6D). During treatment with MC3324, the health status and weight of the mice was monitored (Figure S8F). MC3324 displayed the same anticancer effect in BC specimens, although some differences are due to intra-patient viability. After tissue desegregation, isolated BC cells were treated with MC3324 and a well-known cell death inducer (SAHA); in these samples, MC3324 induced cell death and a reduction of ERα expression, recapitulating the observed effects in MCF7 and BT474 cell lines. The percentage of cell death was lower in healthy cells from isolated neighboring tissue, demonstrating some degree of “tumor selectivity” for MC3324 (Figure 6E). These findings demonstrate that MC3324 is effective in in vivo BC models and can be considered a good candidate in the development of oral drug delivery systems.

## 3. Discussion

BC is a multifactorial disorder representing a major burden for public health and society worldwide [23,24]. As BC is an intricate dynamic disease, drug-resistance phenomena, often still unexplained, are always just around the corner and require a smart approach. Tamoxifen is the most commonly used chemotherapeutic agent for patients with ERα-positive BC, which accounts for almost 70% of all cases. In at least a quarter of all new cases of BC, patients initially responsive go on to develop acquired antiestrogen resistance [25]. In particular, approximately 50% of patients with metastatic disease fail to respond to tamoxifen, and practically all patients with metastasis with initially responsive tumors eventually develop acquired resistance, which becomes the cause of death. Thus, identifying new agents able to overcome resistance in BC is an urgent need. A step forward in BC therapy came about with the development of SERDs (e.g., fulvestrant), antagonists of ERα that also induce its proteasome-mediated degradation. Although fulvestrant is FDA-approved for advanced ERα -positive BC, the poor pharmaceutical properties of this steroid-based SERD have resulted in dose limitations, and, most importantly, not all patients benefit from fulvestrant treatment [26]. A shift from single to multi-target (epigenetic) therapeutic strategies [27,28] appears to be a valuable approach to improving BC management. As for many other malignancies, BC has been correlated with epigenetic alterations that are, crucially and by definition, potentially reversible. Histone demethylases LSD1 and UTX are often co-expressed and co-localized with steroid hormone nuclear receptors [17,29,30,31,32], suggesting a possible role for epigenetic compounds in rearranging steroid hormone signaling and highlighting the possible benefits derived from the use of intrinsically dual targeting molecules with hybrid scaffolds. The idea of using a KDM (LSD1 and UTX) modulator in cancer therapy (acute myeloid leukemia and PC) is not new [33,34,35,36,37,38]. LSD1 and UTX are both part of co-repressor and co-activator complexes and contribute to regulating the activity of specific transcriptional factors including nuclear receptors, therefore their inhibition could be an encouraging strategy to regulate ERα activity in BC [17,29,39,40]. This study focuses on a promising novel KDM inhibitor characterized by a synchronous dual-target structure comprising the active portions of TCP (LSD1 inhibitor) and IOX-1 (JmjC inhibitor), two individually well-known compounds coupled together in a single unit. The molecule, called MC3324, was found in a preliminary screening of KDM inhibitors [18] and it appears to be more effective than its constituent moieties and other known inhibitors used alone or in combination to arrest proliferation and induce cell death in BC. The most attractive feature of MC3324 treatment in BC is the reduction in ERα at mRNA and protein level, in a time- and dose-dependent manner (Figure 1). This effect creates a direct bridge between epigenetic KDM inhibition and hormonal receptor signaling cascade, leading to the activation/downregulation of several biological pathways that have an immediate readout on cell proliferation, migration and death (Figure 2). By acting as an LSD1 and UTX inhibitor, MC3324 induces histone methylation (Figure 5) on residues K4 and k27, regulating ERα recruitment to promoters. Additionally, MC3324 modifies LSD1 and ERα interactors, inducing a response to oxygen-containing substances (Figure 4). The reduction in ERα transcription is also accompanied by turnover of the receptor, leading to a block of pro-proliferative estrogen-mediated stimuli in BC cells after 6 h of treatment. Although the compound does not bind ERα, the overall effect is similar to that obtained with SERDs; in that case the epigenetic rebalance obtained through the LSD1 and UTX inhibition causes ERα downregulation and attenuates hormone signaling, phenocopying the SERD overall effect. MC3324 induces upregulation of death pathways in MCF7 cells, and the RNA-seq profile of treated cells suggests the activation of genes related to tamoxifen overcoming resistance mechanisms (Figure 3). The idea of using a pure epigenetic inhibitor to obtain the same effect as SERD/SERM-acting molecules is also corroborated by the observed induction of cell death in BT474 cells [41,42], which exhibit tamoxifen resistance (Figure 3). In this BC model, MC3324 induced cell death and a strong block in proliferation, accompanied by ERα downregulation. 

The MC3324-mediated anticancer effect was tested in ex vivo and in vivo experiments. Breast specimens obtained from the University of Campania “Luigi Vanvitelli” Department of Surgery, were used to confirm the activity of MC3324 in a system more close mimicking clinical BC. In ex vivo BC cells, MC3324 induced cell death and ERα downregulation. Interestingly, the percentage of cell death in healthy tissues was negligible, recapitulating the effects observed in MCF7, BT474, and HaCaT cell lines (Figure 6 and Figure 3I–L). The anticancer potential of MC3324 was also confirmed in chicken embryos and a mouse model, both engrafted with MCF7 cells. In chicken embryos MC3324 reduced tumor volume and abolished the migratory potential of BC cells compared to tamoxifen. In mice xenograft experiments, MC3324 also proved to be nontoxic, well-tolerated, and effective even if orally administered (Figure 6 and Figure S8). Summarizing the simultaneous inhibition of two KDMs (LSD1 and UTX) could, therefore, be beneficial for BC and, in general, for all tumors in which the hormone receptor system is deregulated, opening the way to epi-based therapies for solid and resistant tumors as well. Indirectly attacking ERα, a key component in cancer progression and maintenance, MC3324 induces cellular reactions leading to cell death. Although the direct inhibition of LSD1 and UTX could have multiple effects, here we focused on intracellular epigenetic regulated cascade which firstly interests hormone signaling. Although LSD1 and UTX are aberrantly regulated in cancer, it is likely not only the expression of both enzymes to determine the efficacy of MC3324, but also their inhibition interference with the hormone-dependent regulatory cascade mediated by ERα. In these settings, the increase in histone methylation impairs the transcription and reduces ERα mRNA and protein level. The absence of ERα impacts on the transcription of thousands of genes that, directly or indirectly, regulate cell proliferation and death. MC3324, therefore, becomes the epigenetic inducer of a regulatory circuit in which hormone signaling pathway is indirectly targeted.

## 4. Materials and Methods 

### 4.1. Chemicals

GSK-LSD1 2HCl, SP2509, ORY-1001, and GSK2879552 2HCl compounds were purchased from Selleckchem (Huston, USA); tamoxifen, TCP, and GSK-J4 from Sigma-Aldrich (St Louis, USA); SAHA from Merck (Kenilworth, N.J., U.S.A). MC3324 was synthesized by Prof. Mai’s group (“Sapienza” University of Rome), as reported in [18]. The MC3324 derivatives MC4379, MC4380, and MC4266 were synthesized as reported in supplementary materials. Compounds were used at concentrations indicated in figures or legends.

### 4.2. Cell Culture 

MCF7 and BT474 cells (purchased from ATCC, (Milano, Italy) were cultured in Dulbecco’s Modified Eagle Medium (DMEM; EuroClone, Milano, Italy), supplemented with 10% heat-inactivated fetal bovine serum (FBS; Sigma-Aldrich, St Louis, USA) antimicrobials (100 U/mL penicillin, 100 μg/mL streptomycin, 250 ng/mL amphotericin-B), and 2 mM L-glutamine (EuroClone). Ex vivo primary cells, isolated by tissue processing of patient biopsies, were grown in DMEM/F12 + 10% FBS. Potential contamination by mycoplasma was monitored and avoided by using MycoAlertTM Assay Control Set (Lonza, Switzerland, Basel) and BM-Cyclin. (Roche, Switzerland, Basel).

### 4.3. Cell Cycle

Cell cycle analysis was performed according to protocol in [43]. 

### 4.4. Histone Extraction

Histones were extracted as reported in [44]. H3K4me2, H3K27me3 (Diagenode pAB-035-050, C15410069, Belgium, Ougrée), H3 (abcam ab2783, Cambridge, United Kingdom), and H4 (abcam ab10158, Cambridge, United Kingdom) were used according to manufacturer’s instructions.

### 4.5. Western Blot Analysis

Detailed protocol is provided in [45]. Primary antibodies used were: ERα (sc-543), ERKs (sc-271269), P53 (sc-126), cyclin D2 (sc-450), ML-IAP (sc-166390), and tubulin (sc-5286) purchased from Santa Cruz (Dallas, USA). LSD1 (ab17721), MAGED1 (ab77045) from Abcam (Cambridge, United Kingdom); UTX (ab33510), caspase 9 (ab9502), caspase 8 (ab9746), BCL2 (ab28725), BAD (ab 5155), P21 (ab 2947), GAPDH (ab 5174) from Cell Signaling (Danvers, Massachusetts). All antibodies were used according to the manufacturer’s instructions. Immunoreactive signals were detected with a horseradish peroxidase-conjugated secondary antibody (GE Healthcare, Chicago, Illinois, USA). Experiments were repeated at least three times. Semi-quantitative analysis was performed using (1.46r, NIH, USA) Relative intensities are reported in figures.

### 4.6. RNA Isolation and Quantitation

Total RNA was isolated, quantified and analyzed by real-time PCR as described in [46]. Real-time PCR was performed using RNA VILO cDNA Synthesis Kit (Invitrogen, Carlsbad, California, USA) to convert RNA into cDNA, and Taq GOLD DNA polymerase (Applied Biosystems, Foster City, California, USA) according to manufacturer’s instructions. ERα primers used: forward 5’ GCTTACTGACCAACCTGGCAG A 3’; reverse 5’ GGATCTCTAGCCAGGCACATTC 3’. GAPDH primers used as normalization control: forward 5’ GGAGTCAACGGATTTGGTCGT 3’; reverse 5’ GCTTCCCGTTCTCAGCCT TGA 3’.

### 4.7. Cellular Thermal Shift Assay(CETSA)

CETSA was performed as reported in [47] and 50 μg of protein extract was loaded on SDS-PAGE, and blotted for LSD1 and UTX. ERKs were used as loading control.

### 4.8. Cell Proliferation Assay

MCF7 cell proliferation was assessed using xCELLigence System (Roche). Cells were seeded in 96-well plates (E-Plate, Roche) at a density of 2 × 105 cells/mL to estimate cellular impedance (a confluence-dependent parameter). Vehicle (DMSO) and MC3324 (25 μM) were added and Cell Index (CI) values were continuously monitored during the whole time of the experiment (70 h), starting from plating time. Measured CI values were visually plotted in a linear graph and histogram.

### 4.9. RNA-Seq and Statistical Analysis 

Detailed protocol for RNA-seq and statistical analysis is reported in Supplementary Materials and Methods.

### 4.10. Co-Immunoprecipitation (Co-IP) and Mass Spectrometry (MS) Analysis 

Detailed methods for IP, High resolution MS, and targeted MS analysis of ER methylation and proteomic data analysis are provided in Supplementary Materials and Methods.

### 4.11. ERα-E2 Radiolabeled Displacement Assay 

Immunoprecipitated ERα was incubated with H3E2 17β-Estradiol radiolabeled with tritium (PerkinElmer, Waltham, Massachusetts, USA) in assay buffer (10 mM Tris-HCl; 1 mM EDTA; 1mM EGTA; 1 mM NaVO3; 10% glycerol; 0.2 mM leupeptin; 10 mg/mL BSA; 1 mM DTT; pH: 7.5) for 3 h. MC3324 was tested after 3 h of co-incubation in escalating doses (25, 50, and 100 µM) and beads were washed several times with the same buffer. Disintegrations per minute were counted by using a liquid scintillation analyzer (Tri-Carb; Packard, PerkinElmer, Waltham, Massachusetts, USA).

### 4.12. Isolation of Cells from Ex Vivo Biopsies

Mammary ex vivo samples were obtained from the “University of Campania “Luigi Vanvitelli’’ Hospital Department of Surgery in collaboration with Dr. Iovino. The use of human derived specimens was allowed by ethics committee (number of protocol 384 of 11/6/2019, entitled: “Epigenetic overcoming of endocrine resistance in breast cancer”). The collected breast samples, included healthy (non-cancerous) and cancerous tissue, were immediately preserved after surgery in FBS-free DMEM and delivered. The samples were weighed and disaggregated by combining mechanical and enzymatic strategies to isolate the cellular component. Enzymatic digestion was achieved with Gibco™ Collagenase Type II used at 600 U/mL/tissue g in FBS-free DMEM and tissue fragments were incubated at 37 °C for 30 min and shaken at 800 rpm with a mixer. Samples were then centrifuged at 500 rpm for 15 min. Supernatants were collected and filtered using 70 μm MACS® filters (Miltenyi Biotech, Bergisch Gladbach, North Rhine-Westphalia, Germany).

### 4.13. Chromatin Immunoprecipitation

MCF7 cells were routinely cultured in DMEM supplemented with 10% FBS at 37 °C and treated for 6 h and 24 h with 25 μM MC3324. Chromatin was harvested as described in [48,49] ChIP experiments were performed using H3K4me2, H3K7me3 (Diagenode, Belgium, Ougrée) and ERα (Santa Cruz, Dallas, USA) antibodies, and isolated DNA was analysed by qPCR. The following primers for promoters were used: ERα forward TGTGCGCCCTAACCAAAGG and reverse TGCTCCCAAAGTAGATAGACCCT; PS2/TFF1 forward GGCCATCTCTCACTATGAATC and reverse GGCAGGCTCTGTTTGCTTAAA; GAPDH forward CAATTCCCCATCTCAGTCGT and reverse GCAGCAGGACACTAGGGAGT.

## Supplementary Materials

The following are available online at https://www.mdpi.com/2072-6694/11/12/2027/s1, Figure S1: MC3324 inhibits LSD1 and UTX in MCF7 cells, Figure S2: Gene Set Enrichment Analysis (GSEA) in MCF7 cells, Figure S3: Comparison of 2933 DE genes with LSD1 binding sites, Figure S4: Comparison of 2933 DE genes with UTX binding sites, Figure S5:LSD1 interaction network changes following MC3324 treatment, Figure S6: Assessment of ERα interactor dynamics following MC3324 treatment and their relation to transcriptomic data, Figure S7: Assessment of LSD1 interactor dynamics following MC3324 treatment and their relation to transcriptomic data, Figure S8: MC3324 does not show toxicity in chicken embryos and mice models, Table S1: List of differentially expressed genes in MCF7 cells after MC3324 treatment, Table S2: List of top up/down-ranked pathways after MC3324 treatment (24 h) in MCF7 cells, Table S3: List of differentially expressed genes containing ERα binding sites after MC3324 treatment, Table S4: List of DE genes containing LSD1 binding sites, Table S5: List of DE genes with UTX binding sites, Table S6: List of interactors of ERα and LSD1 after MC3324 treatment in MCF7 cells (6 h and 24 h, respectively), Table S7: List of peptide transitions after trypsin digestion of ERα protein.
