# Supplementary material for: Inhibition of Histone Demethylases LSD1 and UTX Regulates ERα Signaling in Breast Cancer
Source: Cancers (Basel). 2019 Dec 16;11(12):2027. doi: 10.3390/cancers11122027 (PMC6966629; doi:10.3390/cancers11122027)
Supplement: Supplementary file 1 [file cancers-11-02027-s001.zip › cancers-659480-supplementary/cancers-659480-supplementary-final.pdf]

cancers

Supplementary Materials: Inhibition of Histone Demethylases LSD1 and UTX Regulates ERα Signaling in Breast Cancer

Rosaria Benedetti, Carmela Dell'Aversana, Tommaso De Marchi, Dante Rotili, Ning Qing Liu, Boris Novakovic, Serena Boccella, Salvatore Di Maro, Sandro Cosconati, Alfonso Baldi, Emma Niméus, Johan Schultz, Urban Höglund, Sabatino Maione, Chiara Papulino, Ugo Chianese, Francesco Iovino, Antonio Federico, Antonello Mai, Hendrik G. Stunnenberg, Angela Nebbioso and Lucia Altucci

1. Chemistry

The general synthetic pathway followed for preparation of the final compounds MC4266 (**1**), MC4380 (**2**) and MC4379 (**3**) is depicted in Schematic 1A–C. Derivative **1** was prepared through the coupling reaction between 8-(methoxymethoxy)quinoline-5-carboxylic acid **4,** prepared as described in [1] and *tert*-butyl (2-(4-aminophenyl)propyl)carbamate **6,** synthesized as previously reported [2], performed with 1-hydroxybenzotriazole hydrate (HOBt) and *N*-ethyl-*N*′-(3-dimethylaminopropyl)carbodiimide hydrochloride (EDCI) in the presence of anhydrous triethylamine in dry dimethylformamide (DMF; Schematic 1A). The reaction of the intermediate **8** with 4 N hydrochloric acid in anhydrous 1,4-dioxane yielded the desired *N*-(4-(1-aminopropan-2-yl)phenyl)-8-hydroxyquinoline-5-carboxamide hydrochloride **1** (Schematic 1A). The coupling reaction of the commercially available quinoline-5-carboxylic acid **5** with the intermediate **6** in presence of [bis(dimethylamino)methylene]-1*H*-1,2,3-triazolo[4,5-b]pyridinium 3-oxid hexafluorophosphate (HATU) and *N*,*N*-di*iso*propylethylamine (DIPEA) in dry DMF, followed by the BOC deprotection reaction of the resulting intermediate **9** afforded the final derivative *N*-(4-(1-aminopropan-2-yl)phenyl)quinoline-5-carboxamide hydrochloride **2** (Schematic 1B). Finally, synthesis of *N*-(4-(2-aminocyclopropyl)phenyl)quinoline-5-carboxamide hydrochloride **3** was performed by treating with 4 N hydrochloric acid in dry 1,4-dioxane the intermediate **10**, previously obtained from the coupling reaction in presence of HATU and DIPEA between **5** and the *trans*-*N*-Boc-2-(4-aminophenyl)cyclopropylamine **7**[2] (Schematic 1C).

Schematic 1A–C. Preparation of the final compounds 1–3.[a]

[a]Reagents and conditions: a) 1-Hydroxybenzotriazole hydrate (HOBt), *N*-ethyl-*N*′-(3-dimethylaminopropyl)carbodiimide hydrochloride (EDCI), anhydrous triethylamine (TEA), dry DMF, 0 °C to rt, 28 h (Y = 45%); b) 1-[Bis(dimethylamino)methylene]-1*H*-1,2,3-triazolo[4,5-b]pyridinium 3-oxid hexafluorophosphate (HATU), *N*,*N*-di*iso*propylethylamine (DIPEA), N_2 atmosphere, rt, 3.5–4 h (Y = 53%); c) 4 N hydrochloric acid in anhydrous 1,4-dioxane, dry THF/dry methanol, 0 °C to rt, 22–31 h, (Y = 77–85%).

2. Chemistry Experimental Section

Chemistry. ^{1}H-NMR spectra were recorded at 400 MHz using an AC 400 spectrometer (Bruker); chemical shifts are reported in δ (ppm) units relative to the internal reference compound tetramethylsilane (Me_4Si). Mass spectra were recorded on an API-TOF Mariner instrument (Perspective Biosystem), and samples were injected by a Harvard pump using a flow rate of 5–10 µL/min, infused in an electrospray system. All compounds were routinely checked by thin-layer chromatography (TLC) and ^{1}H-NMR. TLC was performed on aluminum-backed silica gel plates (Merck) with spots visualized by UV light. All solvents were reagent grade and, when necessary, were purified and dried by standard methods. Concentration of solutions after reactions and extractions involved the use of a rotary evaporator operating at reduced pressure of ~20 Torr. Organic solutions were dried over anhydrous sodium sulfate. All chemicals were purchased from Sigma Aldrich or TCI Europe, and were of the highest purity. As a rule, samples prepared for physical and

biological studies were dried in high vacuum over phosphorus pentoxide for 20 h at temperatures ranging from 25 °C to 40 °C, depending on the sample melting point.

General procedure for synthesis of *N*-(4-(1-aminopropan-2-yl)phenyl)-8-hydroxyquinoline-5-carboxamide hydrochloride (1, MC4266), *N*-(4-(1-aminopropan-2-yl)phenyl)quinoline-5-carboxamide hydrochloride (2, MC4380), and *N*-(4-(2-aminocyclopropyl)phenyl)quinoline-5-carboxamide hydrochloride (3, MC4379). Example: N-(4-(1-aminopropan-2-yl)phenyl)quinoline-5-carboxamide hydrochloride (2, MC4380). Compound 9 (39.4 mg, 0.097 mmol, 1.0 equiv.) was dissolved in a mixture of dry tetrahydrofuran (THF)/dry methanol (0.7 mL/1.9 mL) and the solution was stirred at 0 °C. Next, 4 N hydrochloric acid in 1,4-dioxane (1.46 mL, 5.83 mmol, 60 equiv.) was added dropwise and the mixture was allowed to warm at rt. After 25 h, when conversion was complete, the suspension was filtered and washed with dry THF and then with dry diethyl ether to yield 2 (28.3 mg, 85%) as a hygroscopic white solid. ^{1}H-NMR (400 MHz; DMSO) δ ppm: 1.12–1.14 (d, 3H, CHCH_3), 2.64–2.70 (m, 1H, CHCH*H*NH$_2$), 3.00–3.05 (m, 1H, CHC*H*HNH$_2$), 3.41 (br m, 1H, C*H*CH$_3$), 7.26–7.28 (d, 2H, C*H* benzene ring), 7.78–7.85 (m, 3H, C*H* benzene ring and C*H* quinoline ring), 8.00–8.03 (m, 2H, C*H* quinoline ring), 8.09 (m, 3H, NH_2 and NH$_2$ · *H*Cl), 8.33–8.35 (dd, 1H, C*H* quinoline ring), 8.90–8.92 (d, 1H, C*H* quinoline ring), 9.14 (d, 1H, C*H* quinoline ring), 10.78 (s, 1H, CON*H*). MS (ESI) (relative to free amine), m/z: 306 $[M + H]^+$.

N-(4-(1-aminopropan-2-yl)phenyl)-8-hydroxyquinoline-5-carboxamide hydrochloride (1, MC4266). (Y = 77%). ^{1}H-NMR (400 MHz; DMSO) δ ppm: 1.12–1.14 (d, 3H, CHCH_3), 2.65–2.69 (m, 1H, CHC*H*HNH), 2.98–3.02 (m, 1H, CHC*H*HNH), 3.84 (br m, 1H, C*H*CH$_3$), 7.24–7.26 (d, 2H, C*H* benzene ring), 7.31–7.33 (d, 1H, C*H* quinoline ring), 7.76–7.78 (d, 2H, C*H* benzene ring), 7.83 (m, 1H, C*H* quinoline ring), 7.96–7.98 (d, 1H, C*H* quinoline ring), 8.03 (s, 3H, NH_2 and NH$_2$ · *H*Cl), 9.01 (d, 1H, C*H* quinoline ring), 9.05–9.07 (d, 1H, C*H* quinoline ring), 10.55 (s, 1H, CON*H*), 11.30 (br s, 1H, quinoline O*H*). MS (ESI) (relative to free amine), m/z: 322 $[M + H]^+$.

N-(4-(2-aminocyclopropyl)phenyl)-8-hydroxyquinoline-5-carboxamide hydrochloride (3, MC4379). (Y = 78%). ^{1}H-NMR (400 MHz; DMSO) δ ppm: 1.18–1.23 (m, 1H, C*H*H cyclopropane ring), 1.38–1.43 (m, 1H, CH*H* cyclopropane ring), 2.34 (br m, 1H, C*H* cyclopropane ring), 2.80 (br m, 1H, C*H* cyclopropane ring), 7.18–7.20 (d, 2H, C*H* benzene ring), 7.73–7.75 (d, 2H, C*H* benzene ring), 7.80–7.83 (m, 1H, C*H* quinoline ring), 7.98–8.02 (m, 2H, C*H* quinoline ring), 8.31–8.33 (dd, 1H, C*H* quinoline ring), 8.52 (s, 3H, NH_2 and NH$_2$ · *H*Cl), 8.89–8.91 (d, 1H, C*H* quinoline ring), 9.13 (d, 1H, C*H* quinoline ring), 10.74 (s, 1H, CON*H*). MS (ESI) (relative to free amine), m/z: 320 $[M + H]^+$.

Preparation of *tert*-butyl (2-(4-(8-(methoxymethoxy)quinoline-5-carboxamido)phenyl)propyl)carbamate (8). 1-hydroxybenzotriazole (37.8 mg, 0.279 mmol, 1.4 equiv.), *N*-ethyl-*N'*-(3-dimethylaminopropyl)carbodiimide hydrochloride (53.6 mg, 0.279 mmol, 1.4 equiv.), and triethylamine (0.11 mL, 0.759 mmol, 3.8 equiv.) were added in sequence at 0 °C to a mixture of 8-(methoxymethoxy)quinoline-5-carboxylic acid **4** (51.6 mg, 0.220 mmol, 1.1 equiv.) and *tert*-butyl (2-(4-aminophenyl)propyl)carbamate **6** (50 mg, 0.200 mmol, 1.0 equiv.) in dry DMF (1.4 mL), and the resulting solution was stirred at room temperature for 28 h. The reaction was concentrated under reduced pressure, quenched with sodium hydrogen carbonate saturated solution (9 mL) and the aqueous layer was extracted with ethyl acetate (4 × 9 mL). The combined organic phases were washed with sodium hydrogen carbonate saturated solution (2 × 7 mL) and sodium carbonate saturated solution (4 mL) then back-extracted with ethyl acetate (6 mL). The combined organic phases were further washed with brine (2 × 1 mL) and the resulting aqueous layer was back-extracted with ethyl acetate (2 mL). The organic layers were collected together, dried over anhydrous sodium sulfate, filtered, and concentrated *in vacuo*. The resulting crude product was purified by column chromatography on silica gel eluting with a mixture of ethyl acetate/petroleum ether/methanol 5:10:1 to give **8** (42.2 mg, 45%) as an off-white solid. ^{1}H-NMR (400MHz; CDCl3) δ ppm: 1.02–1.04 (d, 3H, CHCH_3), 1.38 (s, 9H, NHCOOC(CH_3)$_3$), 2.58–2.63 (m, 1H, CHCH*H*NH), 2.77–2.81 (m, 1H, CHC*H*HNH), 3.59 (s, 3H, OCH$_2$OCH_3), 3.84 (br m, 1H, C*H*CH$_3$), 4.31 (br m, 1H, N*H*COOC(CH$_3$)$_3$), 5.49 (s, 2H, OCH_2OCH$_3$), 7.14–7.16 (d, 2H, C*H* benzene ring), 7.34–7.36 (d, 1H, C*H* quinoline ring), 7.44–7.47 (m, 1H, C*H* quinoline ring), 7.51–7.53 (d, 2H, C*H* benzene ring), 7.63 (s, 1H,

CON*H*), 7.70–7.72 (d, 1H, C*H* quinoline ring), 8.83–8.85 (d, 1H, C*H* quinoline ring), 8.94 (m, 1H, C*H* quinoline ring). MS (ESI), m/z: 466 $[M + H]^+$.

General procedure for synthesis of the intermediate derivatives 9 and 10. Example: *tert*-butyl (2-(4-(quinoline-5-carboxamido)phenyl)cyclopropyl)carbamate (10). To a solution of quinoline-5-carboxylic acid 5 (38.3 mg, 0.22 mmol, 1.1 equiv.) and *trans*-*N*-Boc-2-(4-aminophenyl)cyclopropylamine 7 (50.0 mg, 0.200 mmol, 1 equiv.) in dry DMF (1 mL) under nitrogen atmosphere, *N*,*N*-di*iso*propylethylamine (0.116 mL, 0.66 mmol, 3.3 equiv.) and [Bis(dimethylamino)methylene]-1*H*-1,2,3-triazolo[4,5-b]pyridinium 3-oxid hexafluorophosphate (84.2 mg, 0.22 mmol, 1.1 equiv.) were added in sequence. After stirring for 4 h at rt, the reaction was concentrated under reduced pressure, quenched with sodium hydrogen carbonate saturated solution (8 mL), and the aqueous layer extracted with ethyl acetate (4 × 8 mL). The organic phase was washed with sodium hydrogen carbonate saturated solution (2 × 4 mL) then back-extracted with ethyl acetate (2 × 2 mL). The combined organic phases were further washed with brine (2 × 1.5 mL) and the resulting aqueous phase was back-extracted with ethyl acetate. The organic layers were collected, dried over anhydrous sodium sulfate, filtered, and concentrated *in vacuo* to give a crude product that was finally purified by column chromatography on silica gel eluting with a mixture of ethyl acetate/petroleum ether/methanol 4:11:1 followed by trituration with a mixture of petroleum ether/diethyl ether 2:1 to provide the derivative 10 (43.6 mg, 53%) as a pure white solid. ^{1}H-NMR (400MHz; DMSO) δ ppm: 1.08–1.11 (m, 2H, CH_2 cyclopropane ring), 1.39 (s, 9H, NHCOOC(CH_3)$_3$), 1.90 (br m, 1H, C*H* cyclopropane ring), 2.60 (br m, 1H, C*H* cyclopropane ring), 7.10–7.12 (d, 2H, C*H* benzene ring), 7.24 (br s, 1H, N*H*COOC(CH_3)$_3$), 7.60–7.64 (m, 1H, C*H* quinoline ring), 7.68–7.70 (d, 2H, C*H* benzene ring), 7.86–7.90 (m, 2H, C*H* quinoline ring), 8.16–8.18 (m, 1H, C*H* quinoline ring), 8.62–8.64 (d, 1H, C*H* quinoline ring), 8.98 (m, 1H, C*H* quinoline ring), 10.56 (s, 1H, CON*H*). MS (ESI), m/z: 404 $[M + H]^+$.

Tert-butyl (2-(4-(quinoline-5-carboxamido)phenyl)propyl)carbamate (9). (Y = 53%). ^{1}H-NMR (400 MHz; CDCl3) δ ppm: 1.02–1.04 (d, 3H, CHCH_3), 1.37 (s, 9H, NHCOOC(CH_3)$_3$), 2.58–2.63 (m, 1H, CHC*H*HNH), 2.77–2.81 (m, 1H, CHCH*H*NH), 3.84 (br m, 1H, C*H*CH_3), 4.32 (br m, 1H, N*H*COOC(CH_3)$_3$), 7.15–7.17 (d, 2H, C*H* benzene ring), 7.42–7.45 (m, 1H, C*H* quinoline ring), 7.54–7.56 (d, 2H, C*H* benzene ring), 7.65–7.69 (t, 1H, C*H* quinoline ring), 7.75–7.77 (m, 2H, C*H* quinoline ring and CON*H*), 8.17–8.19 (d, 1H, C*H* quinoline ring), 8.76–8.78 (d, 1H, C*H* quinoline ring), 8.90 (m, 1H, C*H* quinoline ring). MS (ESI), m/z: 406 $[M + H]^+$.

3. MC3324 Stability

To determine the stability of MC3324 in DMEM (fully complemented with serum and antibiotics as in the main cell-based experiments), the compound was dissolved in DMSO at 25 μM and its concentration was measured by RP-HPLC using an Onyx™ 50 × 2 mm ID C18 column operating at 600 μL/min. The gradient applied was from 1% Solvent B to 70% Solvent B in 10 min. Solvent A was water with added 0.1% trifluoroacetic acid (TFA) and Solvent B was acetonitrile with added 0.1% TFA. The eluate was monitored using a diode array with wavelength set between 200–600 nm. A calibration curve was built by injecting solutions of the compound in DMSO at increasing concentrations between 1.5 μM and 200 μM. After incubation in the medium, the compound concentration was determined at time points between 0 and 60 h. All determinations were in triplicate and are reported as average ± SD.

4. shLSD1 Stable Transfection

shLSD1 vectors (Origene) and the empty vector were used. 1 μg of each vector was transfected into U937 cells using an Amaxa Nucleofector (Lonza), according to the manufacturer’s protocol. After 48 h from transfection, the percentage of GFP-positive cells was determined using cytofluorimetric analysis. shLSD1 and shSCR were maintained in DMEM medium (EuroClone) with 10% heat-inactivated FBS (Sigma-Aldrich), 1% glutamine (EuroClone), 1% penicillin/streptomycin (EuroClone), 0.1% gentamycin (EuroClone) and 500 μg/ml G418 (Gibco) at 37 °C in air containing 5% CO_2 to isolate the positive clones. Downregulation of LSD1 was confirmed by Western blot.

5. Co-Immunoprecipitation (Co-IP)

After induction with MC3324 for 6 hours in MCF7 cells, Co-IP of endogenously expressed ERα protein was performed using whole cell lysate (800 µg) in Co-IP buffer (10mM TRIS pH 7.5, 50 mM NaCl, 10% glycerol, 1 mM EDTA, 1 mM DTT, 10 mM sodium molybdate, 0.2 mM PMSF, 1X Roche Protease Inhibitor Cocktail). Cell lysis was obtained with sonication (Bioruptor, Diagenode). For IP, Protein A/G Plus Agarose (sc-2003; Santa Cruz,) was coated with appropriate ERα antibodie and mixed gently for 2 h at 4 °C. A fraction of the resulting complexes (in triplicate) was washed three times with Wash1 (10 mM TRIS pH 7.5, 50 mM NaCl, 10% glycerol, 1 mM EDTA, 1 mM DTT, 10 mM sodium molybdate, 0.2 mM PMSF, 1X Roche Protease Inhibitor Cocktail), and three times with Wash2 (10 mM TRIS pH 7.5, 50 mM NaCl, 1 mM EDTA, 1 mM DTT, 10 mM sodium molybdate, 0.2 mM PMSF, 1X Roche Protease Inhibitor Cocktail), then denatured and eluted in 2X bromophenol blue as control for IP. The remaining IP complexes were digested with trypsin and eluted as described in [3]. Empore C18-packed stage tips were used to remove any trace of salts [4]. Briefly: three C18 disks were stacked on top of each other and transferred to a pipette tip. Tips were conditioned with methanol and 80% acetonitrile – 0.5% acetic acid in LCMS-grade H_2O (Buffer B), and equilibrated with 0.5% acetic acid in LCMS-grade H_2O (Buffer A). Samples were loaded and washed with Buffer A, then eluted with Buffer B. Peptides were then dried and kept at -80 °C until use. Any remaining detergents from the IP protocol were removed by SP3 protocol [5]. Briefly, dry peptides from the C18 clean-up were incubated with 2 uL of a 50:50 mixture of SeraMag-A and SeraMag-B (Sigma-Aldrich) beads and 200 uL acetonitrile. Beads were then washed once more with pure acetonitrile and eluted by incubation with 2% DMSO in LCMS-grade H_2O. Peptides were then dried and re-suspended in a solution containing 0.1% formic acid in LCMS-grade H_2O.

6. High-Resolution Mass Spectrometry (MS)

Digested peptides derived from IP experiments (in triplicates) were analyzed on a Q-Exactive Plus mass spectrometer coupled to a Proxeon EASY nano-liquid chromatography 1000 system (Thermo Fisher Scientific). A tenth of the total digested peptide mixture volume (corresponding to ~1 ug) was injected into a reverse phase EasySpray analytical column (ID 75 µm × 25 cm C18 2 µm 100 Å particle size; Thermo Fisher Scientific). Gradient was run using LCMS-grade H_2O with 0.1% formic acid (Solvent A) and acetonitrile with 0.1% formic acid (Solvent B) for 110 mins. Gradient was run as follows over a 300 uL/min flow-rate: 5 mins 5% Solvent B, 90 mins 5%–30% Solvent B, 5 mins 30%–95% Solvent B, 10 mins 95% Solvent B. Eluting peptides were subjected to a 1.8 kV spray voltage. High resolution scan was acquired at 70,000 resolution (200 m/z). The 15 most intense ions were fragmented using high-energy induced collision dissociation and fragment spectra were collected at 17,500 resolution. Precursor ions with charge 1 and >6 and with intensities lower than 1.7 × E4 were excluded from triggering fragmentation. Ion accumulation time was set to 100 and 60 msec for MS and MS/MS, respectively. Automatic gain control was set to 1 × E6 for both MS and MS/MS. Dynamic exclusion was enabled and set to 20 sec. Thermo RAW files were acquired using Xcalibur software v3.1.66.10 (Thermo Fisher Scientific).

7. Targeted Mass Spectrometry Analysis of ER Methylation

Targeted selected reaction monitoring (SRM) analyses were performed on a TSQ Vantage Triple Quadrupole mass spectrometer coupled to a Proxeon EASY nano-liquid chromatography II system (Thermo Fisher Scientific). The mass spectrometer was equipped with a nano-electrospray ion source (Thermo Fisher Scientific). Peptides were loaded at a constant 280 bar pressure onto a PicoChip (PCH7515-105H354-FS25; New Objective) analytical column. Solvent A and B used for SRM MS were identical to those used for high-resolution MS. Gradient was run as follows (300 uL/min flow-rate): 1 min 3% Solvent B, 1 min 3%–8% Solvent B, 43 mins 8%–33% Solvent B, 1 min 33%–90% Solvent B, 10 mins 90% Solvent B. The mass spectrometer was operated in SRM mode and both analyzing quadrupoles (Q1 and Q3) were run at 0.7 Da full-width half-maximum resolution. Spray voltage was set to 1.7 kV, dwell time was set to 10 ms, and scan width was set to 0.01 m/z. Collision energies were calculated in Skyline software (v4.1.0.11796; [6]). Thermo RAW files were acquired using Xcalibur

software (v2.1.0.1139). Ion transition lists to be selected and analyzed in SRM mode were generated in Skyline based on the theoretical peptide sequences derived from *in silico* digestion of ERα (Uniprot ID: P03372) using trypsin enzymes on ExPASy (www.expasy.org). Unicity of derived triptic sequences was verified using BLASTp (https://blast.ncbi.nlm.nih.gov). Skyline-generated transition lists for triptic peptides are shown in Supplementary Table 5.

8. Proteomic Data Analysis

High resolution MS data was uploaded to ProteomeXchange via the PRIDE repository [7] with dataset identifier PXD012781. SRM MS data were uploaded to the PASSEL repository [8] with dataset identifier PASS01343. MS analysis RAW files were analyzed by MaxQuant [9] v1.6.0.16. All options were left to default settings except for the feature "Match between runs", which was enabled with default settings. The "proteinGroups" output file was used for data analysis after excluding contaminants, reverse decoy sequences, proteins with a Q-value >0.01, and proteins identified with a minimum of two unique peptides. Protein intensities were then Log10-transformed and a second filtering step was performed. Proteins observed in any negative control experiment were excluded. Statistical and pathway analyses were performed on Log10 protein intensities after filtering out proteins with only one observation per condition (e.g., treatment, no treatment). Welch-corrected t-test followed by Benjamini-Hochberg multiple test correction was performed to determine significant differences between treatment conditions in both ERα and LSD1 immunoprecipitated interaction networks. Statistical tests were performed using GraphPAD (v7.03) and R (v3.4.3). Gene Set Enrichment Analysis (GSEA) was performed using GSEA tools (v2.2.2) with the following parameters: database selected was Gene Ontology Biological Process v5.2, metric used was t-test, scoring method selected was weighted. Heatmaps and GSEA polar scatter plots were generated in R using ComplexHeatmap and ggplot2 packages. Protein interaction maps were visualized in Cytoscape (v3.6.0; www.cytoscape.org) and protein-protein interaction maps for ERα and LSD1 networks were derived from STRING (www.string-db.org) using a 0.40 confidence score cutoff. Thermo RAW files from SRM MS analysis were loaded and analyzed in Skyline. Inspection and manual validation of the results were performed with attention to similarity between library (if available from high-resolution MS) and acquired spectra and retention time windows. Peptide quantities were derived based on the peak areas of each transition group and corrected based on noise areas.

9. RNA-Seq and Statistical Analysis

MCF7 cells were treated with MC3324 (25μM) for 24 hours and total RNA was extracted using the RNEasy Minikit (Qiagen) with on-column DNAseI treatment to degrade any possible DNA contamination. RNA-sequencing libraries were prepared from 300 ng starting RNA material using the KAPA RNA Hyperprep Kit with RiboErase (Roche). Briefly, ribosomal RNA (rRNA) that comprises more than 95% of total RNA is removed using DNA oligos complementary to the rRNA sequence followed by digestion of DNA:RNA duplexes with RNAse H. RNA is then fragmented to an average of 200bp at high temperature in a buffer with magnesium (95 °C for 6 minutes). First and second strand cDNA synthesis is then performed, followed by end repair and A-tailing. Unique adapters are then ligated to each sample (KAPA Single-Indexed Adapter Kit, Set A (30 μM), Roche). Quality of the resulting library is performed to confirm removal of rRNA (by quantitative PCR), and to measure the size of the ligated DNA fragments using the Bioanalyzer High Sensitivity DNA kit (Agilent) and concentration using the Qubit dsDNA HS Assay Kit (Life Technologies). Sequencing was performed on the NextSeq500 (Illumina) using the TG NextSeq® 500/550 High Output Kit v2 (75 cycles) (Illumina). RNA-seq reads were aligned using Bowtie mapper [10] against the GRCh37.72 reference human transcriptome. The reads with mapping quality score <15 were removed from further analysis. Mapped transcripts were quantified using mmseq pipeline to determine gene expression levels as both 'unique reads per gene' and 'reads per kilobase per million (RPKM)' [11]. Unique reads were used to identify differentially expressed genes using DESeq (20979621). The RPKM values were used to compare the difference of gene expression between untreated and MC3324-treated cells. The significantly differentially expressed (DE) genes were determined at a

threshold of FDR ≤0.001 and the absolute value of log2Ratio ≥2. Gene set enrichment analysis (GSEA) [12] was performed against MSigDB cancer hallmark database [13]. The data discussed in this publication have been deposited in NCBI's Gene Expression Omnibus and are accessible through GEO Series accession number GSE130067 (https://www.ncbi.nlm.nih.gov/geo/query/acc.cgi?acc=GSE130067).

10. ERα, LSD1 and UTX Binding Site Intersection with RNA-Seq

ChIP-seq peaks for ERα, LSD1 and UTX transcription factors were retrieved from the Gene Expression Omnibus (GEO) repository. Specifically, we retrieved the ChIP-seq peaks from the following datasets: GSE119057 for ERα treated with estra-2-diol for 30′, GSE104755 for LSD1 and GSE96996 for UTX. The ChIP peaks were annotated using the Bioconductor package ChIPseeker. The peaks annotation was performed by the use of the TxDb.Hsapiens.UCSC.hg19.knownGene and org.Hs.eg.db Bioconductor packages. Graphics and images were performed through the use of ChIP seeker and ggplot2 R packages. Arithmetic computations at genome level were performed using the BEDtools toolset v2.26.0.

11. Docking Calculations

The X-ray structure of UTX (PDB code 6G8F; [14]) was retrieved from the Protein Data Bank and prepared for docking calculations of MC3324 with the Maestro Protein Preparation Wizard graphical user interface which assigns bond orders, adds hydrogen atoms, and generates appropriate protonation states. The co-crystal manganese metal was replaced by a Fe^{2+} ion; all other co-crystal non-protein molecules were removed with the exception of the two waters chelating the metal ion. The MC3324 ligand was prepared with the builder module within Maestro (http://www.schrodinger.com/ 2018.3) and then geometrically optimized with Macromodel. Docking studies were performed using the Glide tool. The docking grid box was centered on the residues lining the Fe^{2+}, with a grid box dimension equal to 24 Å × 24 Å × 24 Å. Finally, docking runs were carried out using the standard precision method. Images were rendered with UCSF Chimera software [15].

12. In vivo INOVOTION Model

A chick embryo tumor growth and metastasis assay (INOVOTION) was performed as previously described [16]. Briefly, fertilized White Leghorn eggs (SFPA, Hendrix Genetics) were incubated at 38 °C with 60% relative humidity for nine days. The chorioallantoic membrane (CAM) was then dropped, and a 1 cm^2 window was cut in the eggshell above the CAM (at day 9). MCF7 cells were harvested by trypsinization, washed with complete medium, and a 50 μL inoculum of one million cells was added directly onto the CAM of each egg. Eggs were randomly allocated into four groups of 16–17 eggs to obtain sufficient surviving embryos at the end of the experiments. At day 10, when tumors began to be detectable, eggs were treated every two days for 10 days by dropping 100 μL of either 100 μmol OH-Tam, 25 μmol and 50 μmol MC3324, or vehicle (0.1% DMSO in PBS) onto the tumor. At day 19, the upper portion of the CAM was removed and transferred to PBS, and the tumors were carefully cut away from normal CAM tissue and weighed. In parallel, a 1 cm^2 portion of the lower CAM was collected to evaluate the number of MCF7 cells. To count these cells, genomic DNA was extracted, and qPCR analysis was performed using primers specific for genomic human Alu/repetitive sequences. Finally, the toxicity of the treatment was evaluated by scoring the number of dead embryos.

13. Tumor Xenograft Experiments

Hsd:Athymic Nude-Foxn1 Nu/nu 6–7-week-old recipient mice (Envigo) were transplanted with 5,000,000 MCF7 cells after E2 pellet (Belma) supplement (one week). At tumor onset (successful tumor engraftment = 60%), mice were treated orally with 50 mg/Kg MC3324, two days/week for 16 days. Mice were daily monitored for symptoms of disease (ruffled coat, hunched back, weakness and reduced motility). Tumor masses were measured and at the end of scheduled treatment with MC3324 mice were euthanized by cervical dislocation and tumors isolated. Data reports pilot tumor growth

using 8 animals for group. The average volume of tumor was calculated as V = (W(2) × L)/2 for caliper measurements, here V is tumor volume, W is tumor width, L is tumor length. Animal experiments were performed in compliance with institutional guidelines and regulations and after approval from the institutional review board. Approval number identifying the protocol is 626/2015-PR.

14. Immunohistochemistry in Chicken Embryos and Mice Tumors

MCF7-derived masses were fixed in 10% neutral buffered formalin, routinely processed and embedded in paraffin blocks. Four-μm-thick sections obtained from these blocks were then immunostained with primary rabbit monoclonal antibodies against Ki-67, ERα and E-cadherin (chicken embryos) and against Ki-67, ERα, H3K4me2 and H3K27me3 (mice tumors) using a standard immunoperoxidase protocol (BA-1000 Biotinylated Goat anti-Rabbit IgG Antibody and PK-6100 Vectastain Elite ABC kit; Vector Laboratories) followed by diaminobenzidine chromogenic reaction (SK-4100 DAB Peroxidase Substrate Kit; Vector Laboratories). Serial sections incubated with normal goat serum instead of the primary antibody served as negative control.

15. Acute Toxicity in Mice

MC3324 underwent an initial *in vitro* evaluation study followed by a Stage 1 pharmacokinetics (PK) study using a dose of 50 mg/kg MC3324 p.o. The *in vitro* evaluation study consisted of the following steps:

- QC of compound (identity, purity and chemical stability)
- Determination of kinetic solubility of the compound in buffer
- Development of an MRM method.

The Stage 1 PK study consisted of the following steps:

- Preparation of dose solution for *in vivo* experiments
- Administration of MC3324 to mice (two male mice orally by gavage); blood sampling at 30, 60 and 180 min). Following the last blood sample, the animals were euthanized and plasma was collected from the bladder.
- The health status of mice was documented at sampling times.
- Bioanalysis of plasma samples by liquid chromatography (LC)-MS/MS.

Mass spectrometry: Instrument: ACQUITY UPLC-TQD (Waters)

LC method**:** Column: BEH C18 1.7 μm (Waters), Mobile phase: A, 0.1% formic acid in water; B, 100% acetonitrile. Gradient starting at 3% B and switching to 95% B after 1.1 min, and to 97% B after 1.5 min. Total time: 2.5 min. Flow: 0.4 ml/min.

MRM method for MC3324**:** Ionization mode: ES+, Parent (m/z): 320.20, Daughter (m/z): 172.14, Cone voltage: 40 V, Collision voltage: 24 V

MRM method for propranolol (internal standard): Ionization mode: ES+, Parent (m/z): 260.2, Daughter (m/z): 116.2, Cone voltage: 40 V, Collision voltage: 22 V

Final formulation: 5 mg/ml MC3324 in 7% DMSO, 35% HPβCD in DPBS, pH 6.5.

16. Concentration of MC3324 in Plasma Samples

Plasma sample preparation: 15 μl of each plasma sample and standards were added to 75 μl (5 volumes) cold acetonitrile with 1 μM propranolol in a 96-well plate. Plate was left for 20 min equilibration on a shaker table and then centrifuged for 15 min at 2800 rpm. 40 μl of the supernatants was transferred to wells containing 80 μl distilled water in another 96-well plate. LC-MS/MS experiments were run. The analysis of each sample was performed in duplicate.

Standard sample preparation: MC3324 standard samples in inactive mouse plasma were prepared (40 μM and 10 μM). The 10 μM standard sample was used to perform a serial dilution in steps by a factor of 4 using inactive mouse plasma to prepare the other five standard samples. The standard samples were prepared in the same way as plasma samples.

Health status of animals following administration: No abnormal clinical symptoms were observed for the two mice that were administered 50 mg/kg MC3324 p.o.

17. Statistical Analysis

Data were presented as the mean ± SD of biological triplicates. Differences between treatment groups and controls were compared using one-way analysis of variance (ANOVA) and Dunnett's multiple-comparison test. Differences between groups were considered to be significant at a p-value of <0.05 (or <0.01 as reported in figures). Statistical analyses were performed using GraphPad Prism 6.0 software (GraphPad Software).

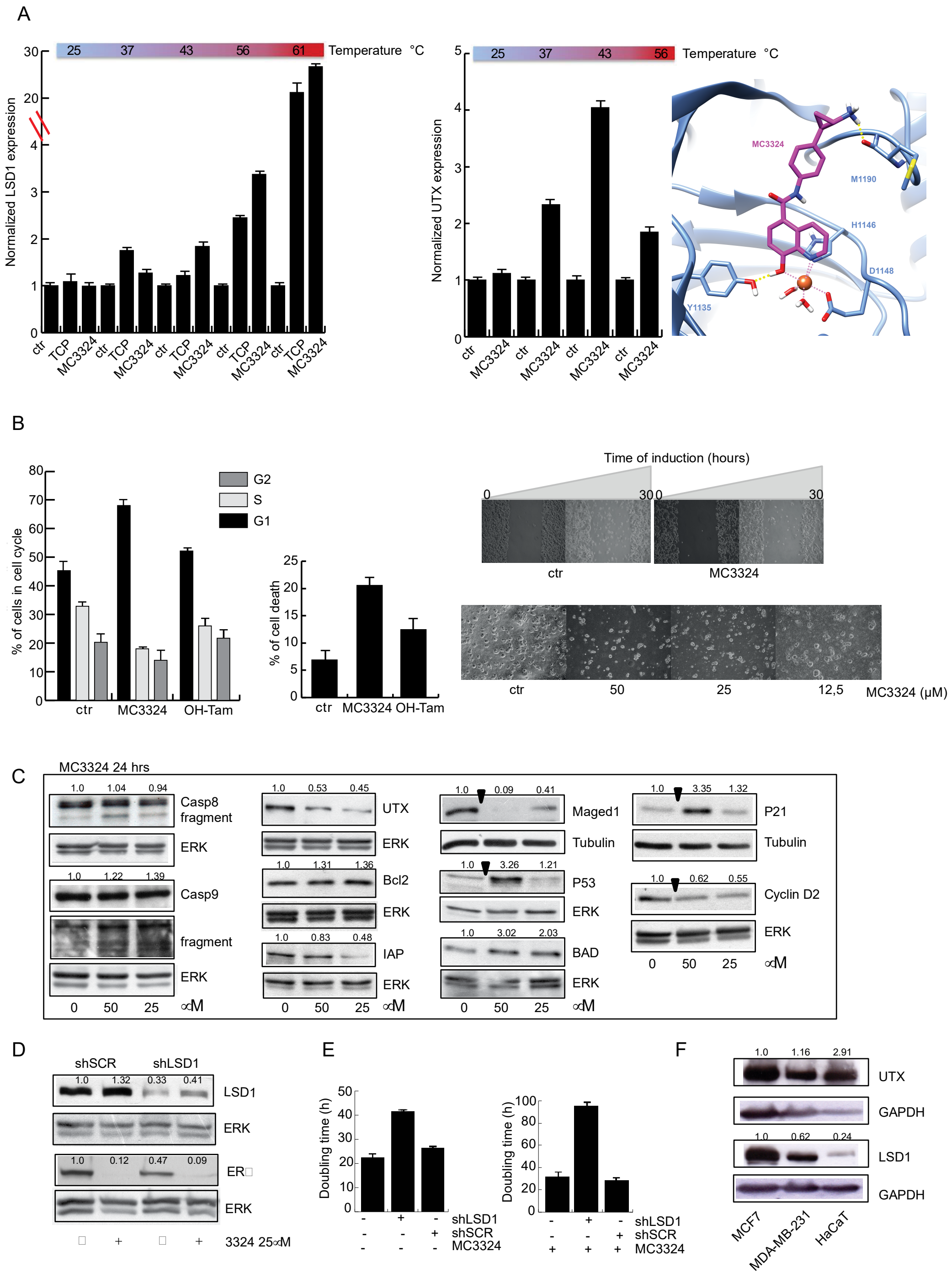

Figure S1. MC3324 inhibits LSD1 and UTX in MCF7 cells.(**A**) CETSA assay for LSD1 (left) and UTX (right) after MC3324 treatment (1 h at 25 µM). Binding mode of MC3324 in UTX binding site as calculated by docking experiments is also reported. The ligand is represented as magenta sticks while the protein is depicted as blue ribbons and sticks. H-bonds and chelation interactions are outlined by dashed yellow and purple lines, respectively. The 8-hydroxyquinoline moiety is able to chelate the Fe^{2+} ion while the protonated nitrogen of the TCP portion forms a charge-reinforced H-bond with M1190 backbone CO of the protein. (**B**) Arrest of proliferation, induction of cell death, colony formation inhibition and reduction in migration after MC3324 treatment in MCF7cells (25 µM at indicated times). (**C**) MC3324 (24 h) regulates proteins involved in BC pathways. (**D**) Downregulation of LSD1 decreases ERα with a slight effect on histone modifications. (**E**) shLSD1 reduces MCF7 proliferative index. The standard deviations are derived from independent replicates. (**F**) UTX and LSD1 basal level expression in MCF7, MDA-MB-231 and HaCaT cell lines.

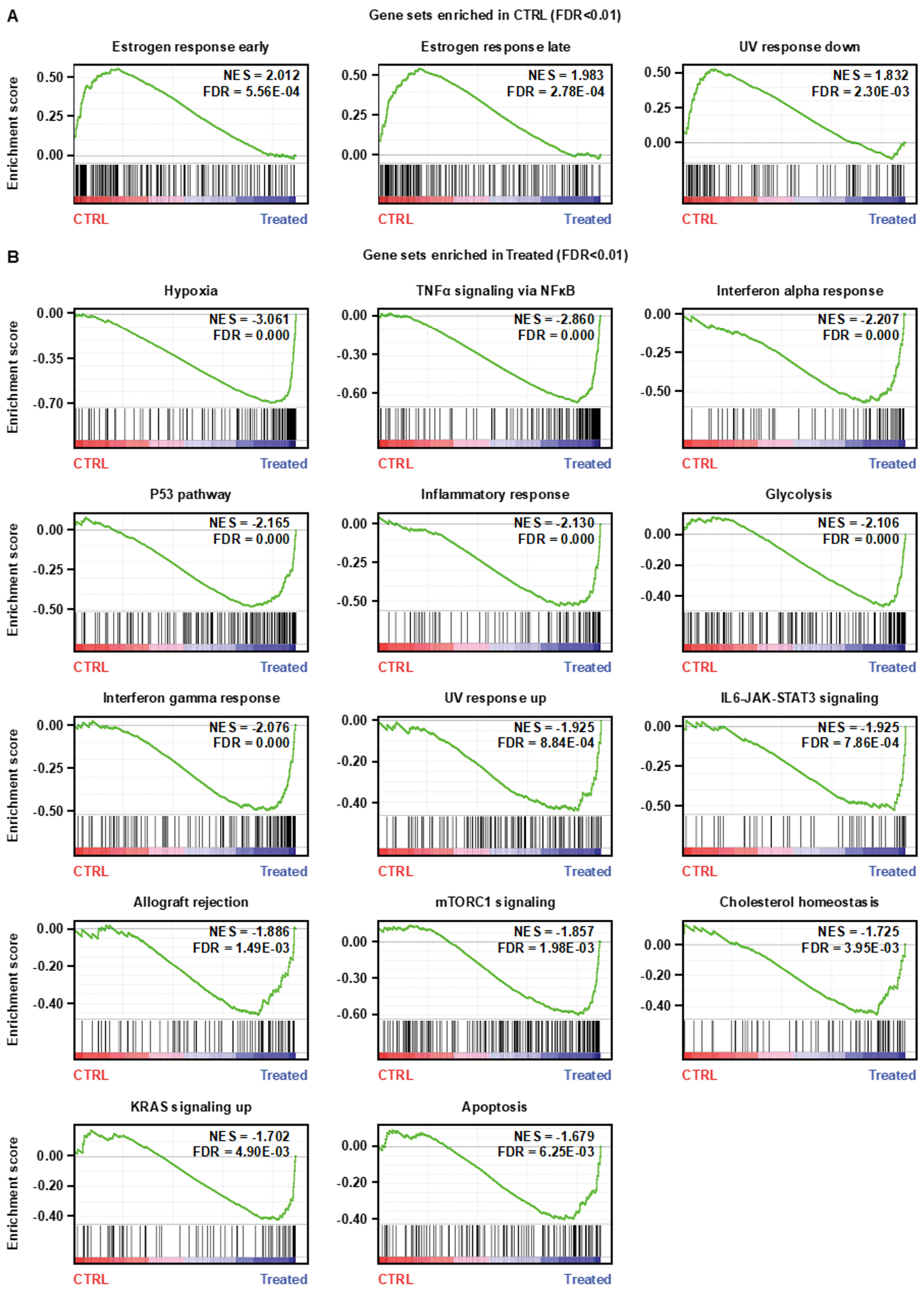

Figure S2. Gene Set Enrichment Analysis (GSEA) in MCF7 cells (**A**) Gene sets enriched in CTRL and (**B**) in MC3324 treated MCF7 cells for 24h at the concentration of 25μM. The gene expression data generated by RNA-seq was analyzed using GSEA. Highly, significant enriched gene-sets are shown here (FDR<0.01). In every thumbnail, the green curve represents the evolution of the density of the genes identified in the RNA-seq. The False Discovery Rate (FDR) is calculated by comparing the actual data with 1000 Monte-Carlo simulations. The NES (Normalized Enrichment Score) computes the density of modified genes in the dataset with the random expectancies, normalized by the number of genes found in a given gene cluster, to take into account the size of the cluster.

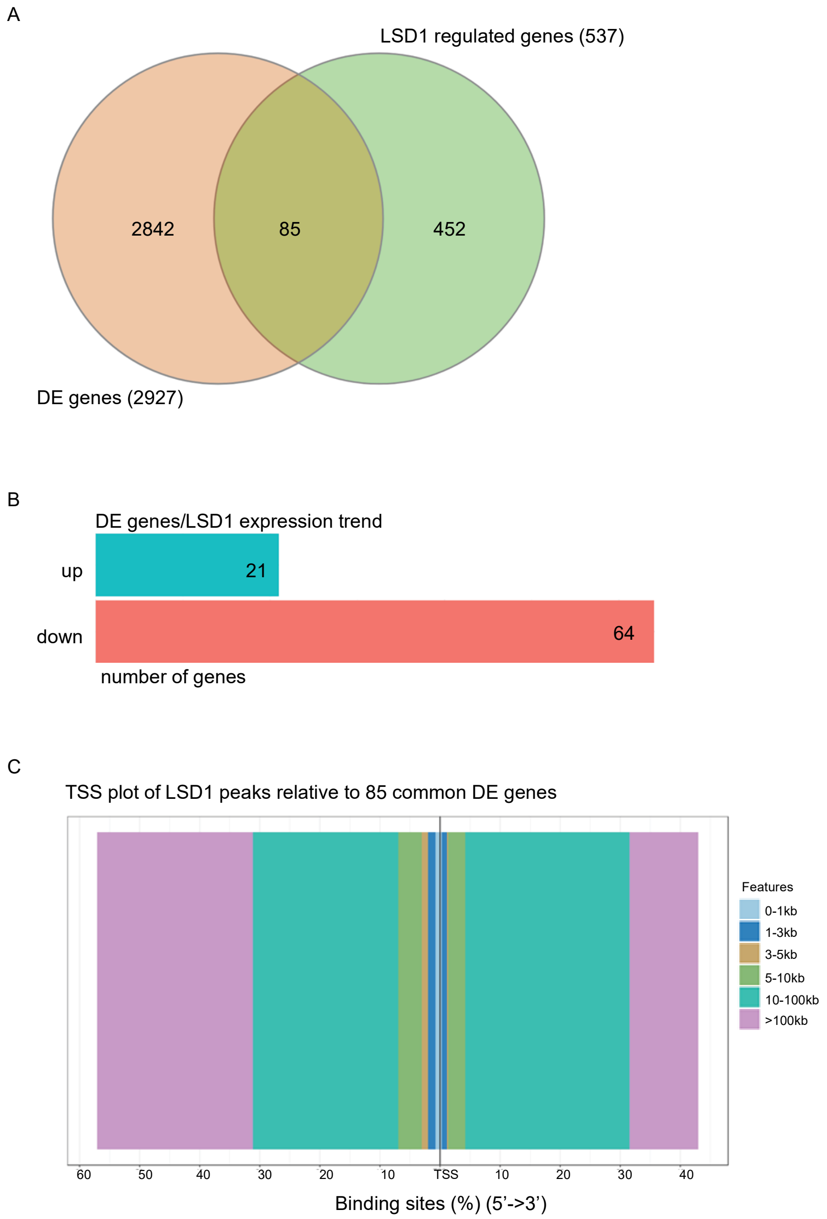

Figure S3. Comparison of 2933 DE genes with LSD1 binding sites. (**A**) Venn diagram summarizing results relative to deregulated mRNA co-associated with LSD1 binding sites. (**B**) Barplot of up/down-regulated genes associated with LSD1 binding sites. 85 genes are differentially expressed (up: 21; down: 64) and show LSD1 binding site in MCF7 after the treatment with MC3324 for 24 hours. (**C**) TSS plot of 85 regulated genes displays the number of reads that map around the transcription start site of genes within defined regions (from 0kb to >100kb).

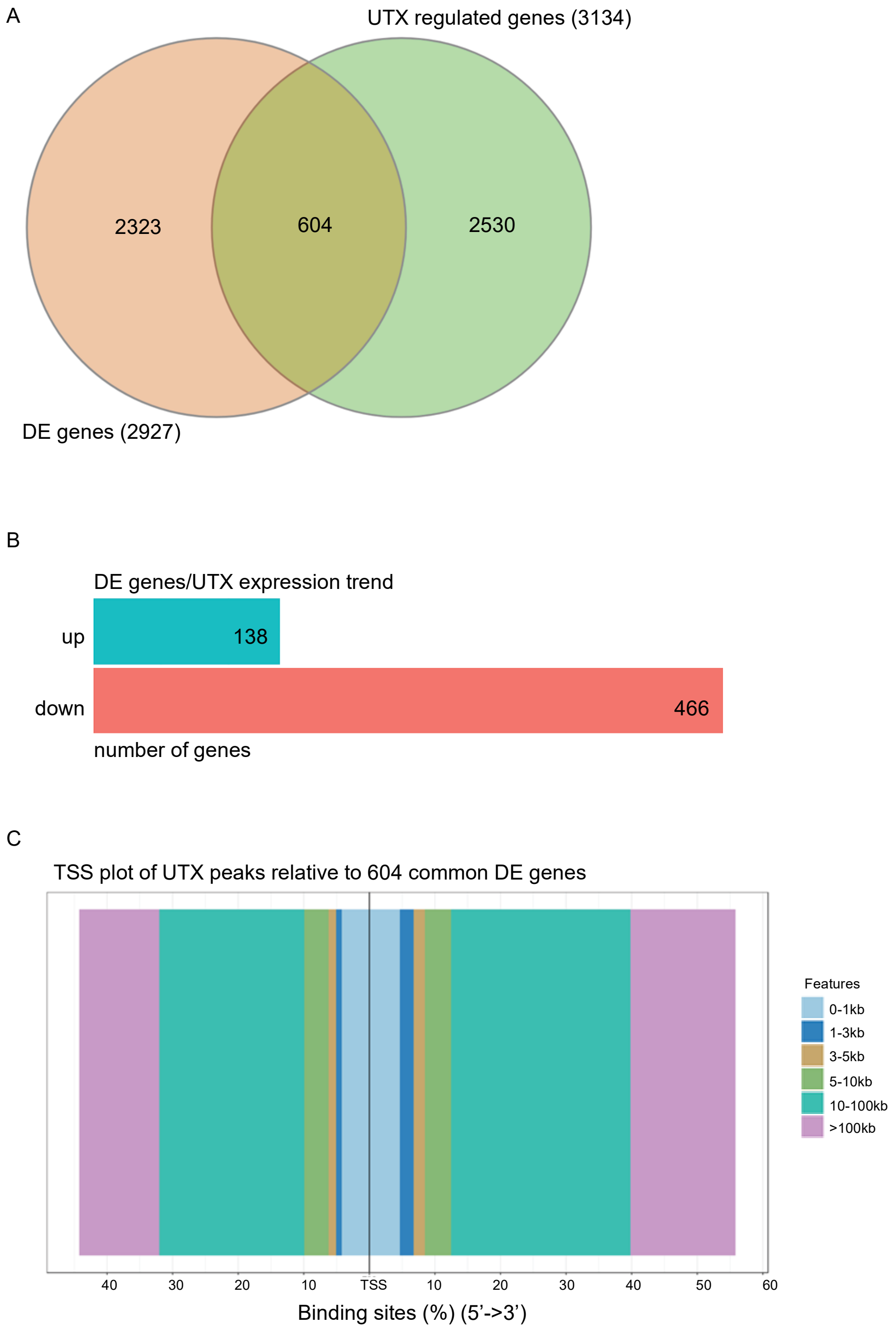

Figure S4. Comparison of 2933 DE genes with UTX binding sites. (**A**) Venn diagram summarizing results relative to deregulated mRNA co-associated with UTX binding sites. (**B**) Barplot of up/down-regulated genes associated with UTX binding sites. 604 genes are differentially expressed (up: 138; down: 466) and show UTX binding site in MCF7 after the treatment with MC3324 for 24 hours. (**C**) TSS plot of 604 regulated genes displays the number of reads that map around the transcription start site of genes within defined regions (from 0kb to >100kb).

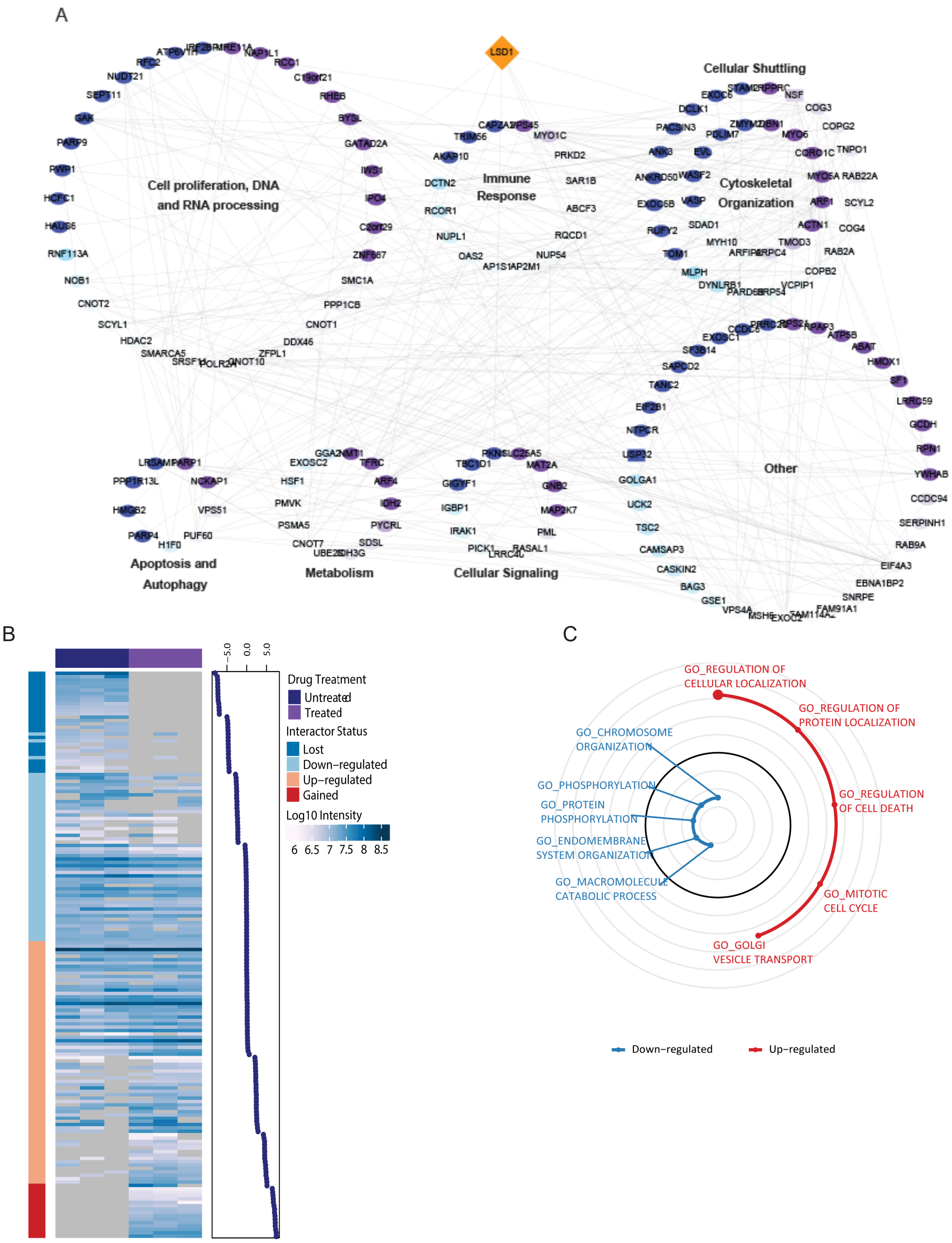

Figure S5. LSD1 interaction network changes following MC3324 treatment. (**A**) Proteins identified by LSD1 pull-down experiments after treatment with MC3324 (25 μM for 24 h) were annotated and clustered based on Gene Ontology Biological Process (GOBP) terms and visualized as a STRING (www.string-db.org) network in Cytoscape. Nodes represent identified proteins while edges represent interactions derived from the STRING database. Node color code: pull-down target (orange), up-regulated interactors (purple), down-regulated interactors (light blue). (**B**) Heatmap of LSD1 interactors (negative and positive Log2 Ratio) after MC3324 treatment. GSEA analysis was performed to assess which pathways (**C**) displayed significant regulation following MC3324 treatment.

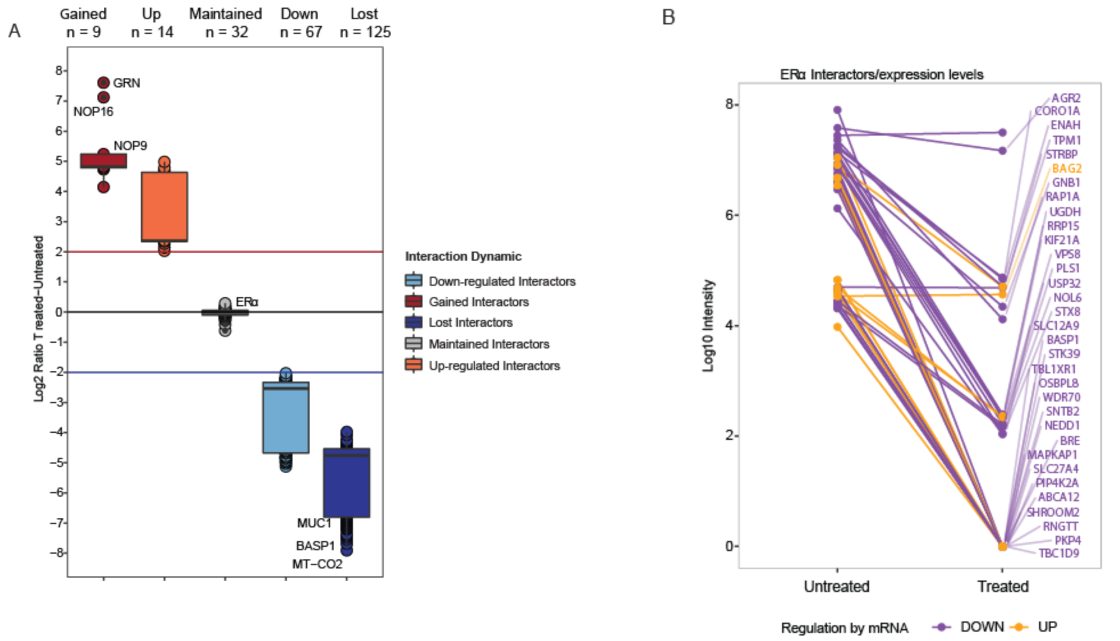

Figure S6. Assessment of ERα interactor dynamics following MC3324 treatment and their relation to transcriptomic data. Box plots (**A**) of ERα protein interaction network distribution after treatment with MC3324 (25μM for 6 h) revealed a large variation in protein dynamics, with many proteins either down-regulated or lost following treatment. For the majority of identified proteins from our pull-down experiments, ERα interactor dynamics matched transcriptional data obtained under the same conditions in MCF7 cells, where down-regulated transcripts showed a similar trend at protein level (**B**). For 34 (33 down and 1 up) proteins, RNA-seq and MS data show the same trend.

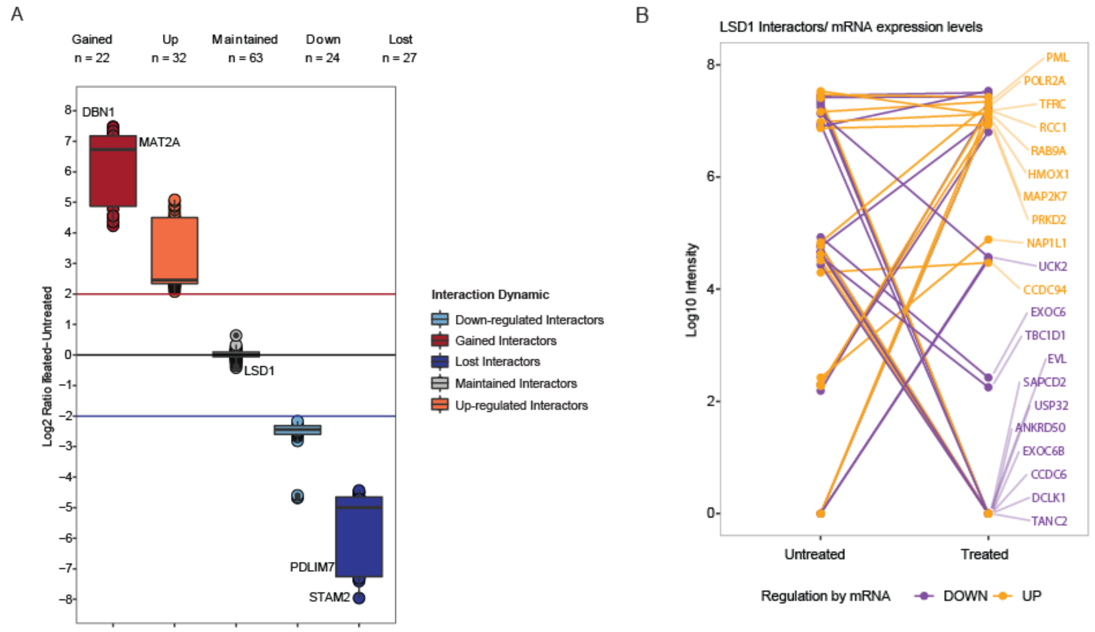

Figure S7. Assessment of LSD1 interactor dynamics following MC3324 treatment and their relation to transcriptomic data. Box plots (**A**) of LSD1 protein interaction network distribution after treatment with MC3324 (25 μM for 24 h). Some identified proteins from LSD1 pull-down experiments match with transcriptional data for MCF7 cells (**B**). For 21 (11 down and 10 up) out of 36 proteins (58.3%), RNA-seq and MS data show the same trend.

A

	number	Tumor weight (mg)	SD	SEM	% of tumor regression
**Veicle**	16	9,997	3,332	0,86	N/A
**4OH-T**	17	7,767	2,155	0,576	22,31
**MC3324**	17	6.795	2.218	0.555	32.03

B

	Total	Alive	Dead	% alive	% dead
**Veicle**	16	16	0	100	0
**4OH-T**	17	17	0	100	0
**MC3324**	17	117	0	100	0

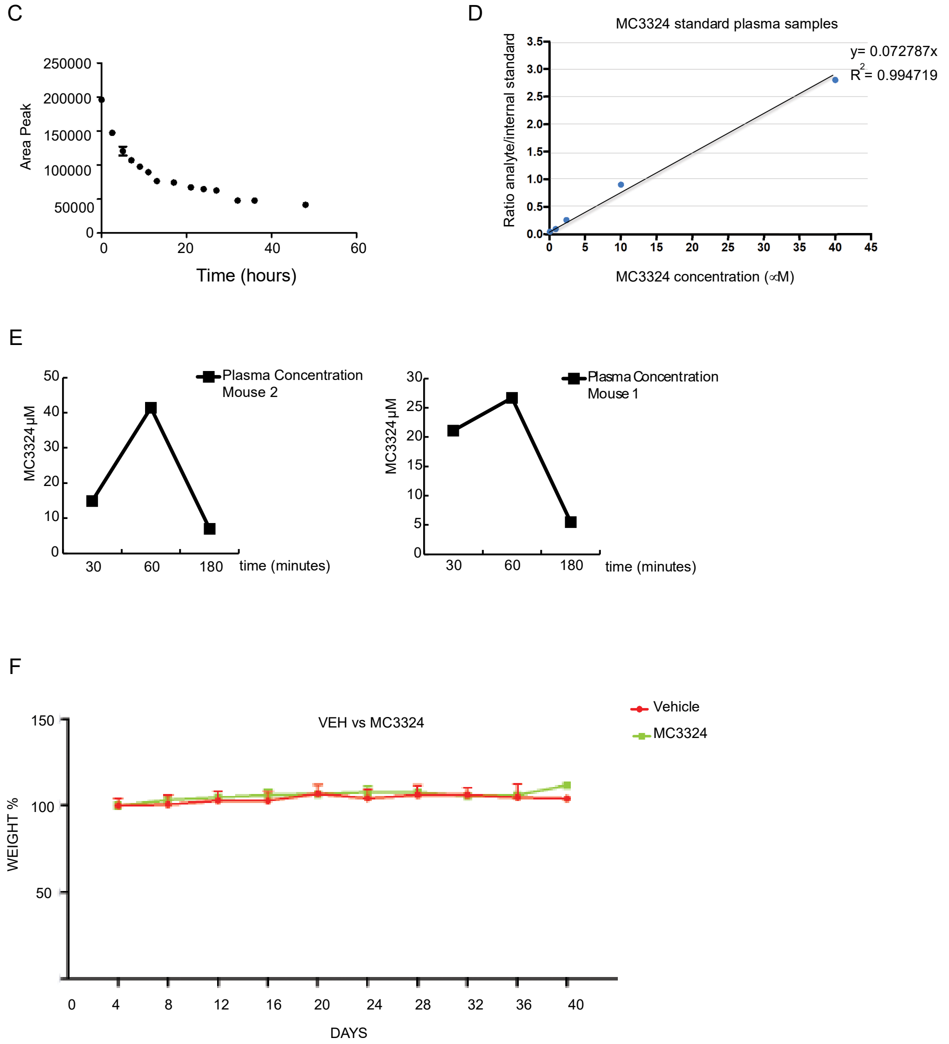

Figure S8. MC3324 does not show toxicity in chicken embryos and mice models. (**A**) Table summarizes % of tumor weight and tumor regression in chicken embryos after treatment with MC3324 as reported in main Figure 6. (**B**) Table with % of dead and alive chicken embryos after treatment. (**C**) MC3324 stability in cell medium (DMEM plus 10% FBS). (**D**) MC3324 concentration in standard plasma samples. (**E**) MC3324 plasma concentration in mice after p.o administration. Sampling was performed after 30, 60 and 180 min. (**F**) % of weight variation in MC3324- and vehicle-treated mice during the time.

Table S1. List of differentially expressed genes in MCF7 cells after MC3324 treatment.
Table S2. List of top up/down-ranked pathways after MC3324 treatment (24 h) in MCF7 cells.
Table S3. List of differentially expressed genes containing ER*α* binding sites after MC3324 treatment.
Table S4. List of DE genes containing LSD1 binding sites.

Table S5. List of DE genes with UTX binding sites
Table S6. List of interactors of ERα and LSD1 after MC3324 treatment in MCF7 cells (6 h and 24 h, respectively).
Table S7. List of peptide transitions after trypsin digestion of ERα protein.

References

1. Rotili, D.; Tomassi, S.; Conte, M.; Benedetti, R.; Tortorici, M.; Ciossani, G.; Valente, S.; Marrocco, B.; Labella, D.; Novellino, E.; et al. Pan-histone demethylase inhibitors simultaneously targeting Jumonji C and lysine-specific demethylases display high anticancer activities. *J Med Chem* **2014**, *57*, 42–55, doi:10.1021/jm4012802.
2. Binda, C.; Valente, S.; Romanenghi, M.; Pilotto, S.; Cirilli, R.; Karytinos, A.; Ciossani, G.; Botrugno, O.A.; Forneris, F.; Tardugno, M.; et al. Biochemical, structural, and biological evaluation of tranylcypromine derivatives as inhibitors of histone demethylases LSD1 and LSD2. *J Am Chem Soc* **2010**, *132*, 6827–6833, doi:10.1021/ja101557k.
3. Hubner, N.C.; Mann, M. Extracting gene function from protein-protein interactions using Quantitative BAC InteraCtomics (QUBIC). *Methods* **2011**, *53*, 453–459, doi:10.1016/j.ymeth.2010.12.016.
4. Wisniewski, J.R.; Zougman, A.; Nagaraj, N.; Mann, M. Universal sample preparation method for proteome analysis. *Nat Methods* **2009**, *6*, 359–362, doi:10.1038/nmeth.1322.
5. Hughes, C.S.; Foehr, S.; Garfield, D.A.; Furlong, E.E.; Steinmetz, L.M.; Krijgsveld, J. Ultrasensitive proteome analysis using paramagnetic bead technology. *Mol Syst Biol* **2014**, *10*, 757, doi:10.15252/msb.20145625.
6. MacLean, B.; Tomazela, D.M.; Shulman, N.; Chambers, M.; Finney, G.L.; Frewen, B.; Kern, R.; Tabb, D.L.; Liebler, D.C.; MacCoss, M.J. Skyline: an open source document editor for creating and analyzing targeted proteomics experiments. *Bioinformatics* **2010**, *26*, 966–968, doi:10.1093/bioinformatics/btq054.
7. Vizcaino, J.A.; Cote, R.G.; Csordas, A.; Dianes, J.A.; Fabregat, A.; Foster, J.M.; Griss, J.; Alpi, E.; Birim, M.; Contell, J.; et al. The PRoteomics IDEntifications (PRIDE) database and associated tools: status in 2013. *Nucleic Acids Res* **2013**, *41*, D1063–1069, doi:10.1093/nar/gks1262.
8. Farrah, T.; Deutsch, E.W.; Kreisberg, R.; Sun, Z.; Campbell, D.S.; Mendoza, L.; Kusebauch, U.; Brusniak, M.Y.; Huttenhain, R.; Schiess, R.; et al. PASSEL: the PeptideAtlas SRMexperiment library. *Proteomics* **2012**, *12*, 1170–1175, doi:10.1002/pmic.201100515.
9. Cox, J.; Mann, M. MaxQuant enables high peptide identification rates, individualized p.p.b.-range mass accuracies and proteome-wide protein quantification. *Nat Biotechnol* **2008**, *26*, 1367–1372, doi:10.1038/nbt.1511.
10. Langmead, B.; Trapnell, C.; Pop, M.; Salzberg, S.L. Ultrafast and memory-efficient alignment of short DNA sequences to the human genome. *Genome Biol* **2009**, *10*, R25, doi:10.1186/gb-2009-10-3-r25.
11. Turro, E.; Su, S.Y.; Goncalves, A.; Coin, L.J.; Richardson, S.; Lewin, A. Haplotype and isoform specific expression estimation using multi-mapping RNA-seq reads. *Genome Biol* **2011**, *12*, R13, doi:10.1186/gb-2011-12-2-r13.
12. Subramanian, A.; Tamayo, P.; Mootha, V.K.; Mukherjee, S.; Ebert, B.L.; Gillette, M.A.; Paulovich, A.; Pomeroy, S.L.; Golub, T.R.; Lander, E.S.; et al. Gene set enrichment analysis: a knowledge-based approach for interpreting genome-wide expression profiles. *Proc Natl Acad Sci U S A* **2005**, *102*, 15545–15550, doi:10.1073/pnas.0506580102.
13. Liberzon, A.; Subramanian, A.; Pinchback, R.; Thorvaldsdottir, H.; Tamayo, P.; Mesirov, J.P. Molecular signatures database (MSigDB) 3.0. *Bioinformatics* **2011**, *27*, 1739–1740, doi:10.1093/bioinformatics/btr260.
14. Esposito, C.; Wiedmer, L.; Caflisch, A. In Silico Identification of JMJD3 Demethylase Inhibitors. *J Chem Inf Model* **2018**, *58*, 2151–2163, doi:10.1021/acs.jcim.8b00539.
15. Pettersen, E.F.; Goddard, T.D.; Huang, C.C.; Couch, G.S.; Greenblatt, D.M.; Meng, E.C.; Ferrin, T.E. UCSF Chimera—a visualization system for exploratory research and analysis. *J Comput Chem* **2004**, *25*, 1605–1612, doi:10.1002/jcc.20084.
16. Mikaelian, I.; Malek, M.; Gadet, R.; Viallet, J.; Garcia, A.; Girard-Gagnepain, A.; Hesling, C.; Gillet, G.; Gonzalo, P.; Rimokh, R.; et al. Genetic and pharmacologic inhibition of mTORC1 promotes EMT by a TGF-beta-independent mechanism. *Cancer Res* **2013**, *73*, 6621–6631, doi:10.1158/0008-5472.CAN-13-0560.

© 2019 by the authors. Submitted for possible open access publication under the terms and conditions of the Creative Commons Attribution (CC BY) license (http://creativecommons.org/licenses/by/4.0/).
