# Supplementary figures and images for: Inhibition of Histone Demethylases LSD1 and UTX Regulates ERα Signaling in Breast Cancer
Source: Cancers (Basel). 2019 Dec 16;11(12):2027. doi: 10.3390/cancers11122027 (PMC6966629; doi:10.3390/cancers11122027)

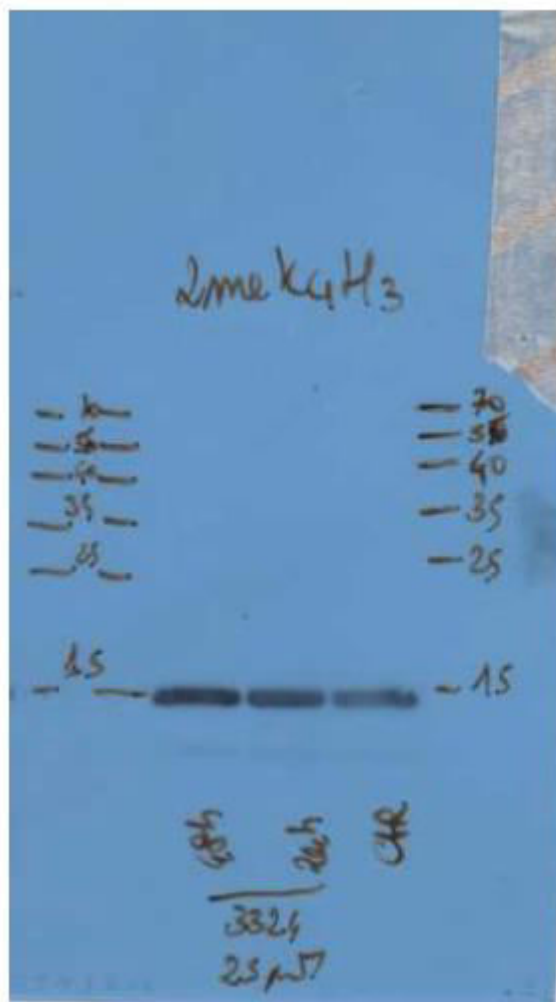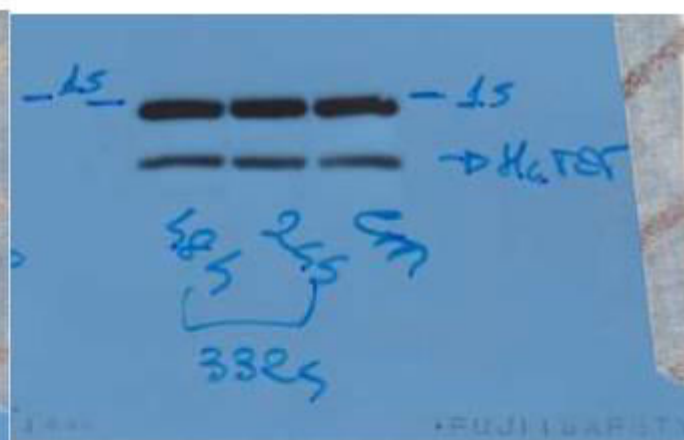

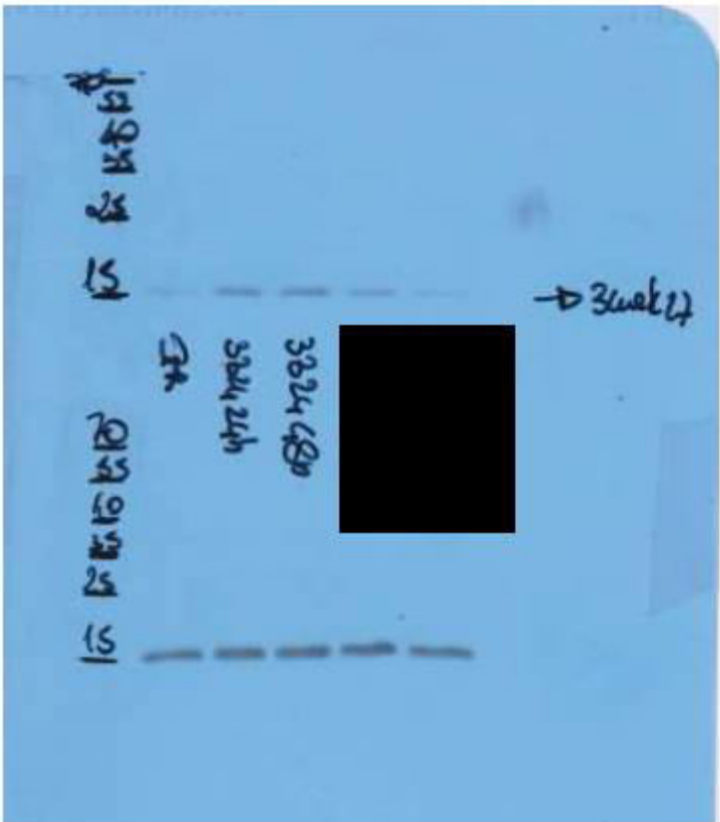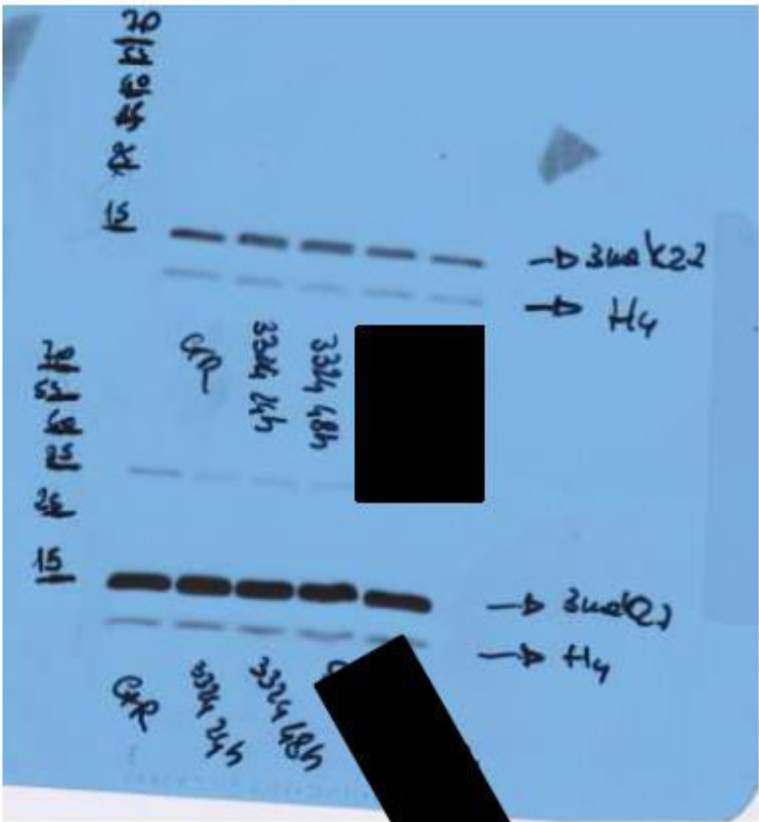

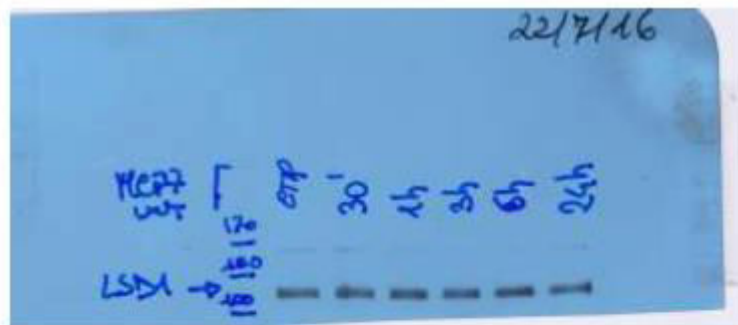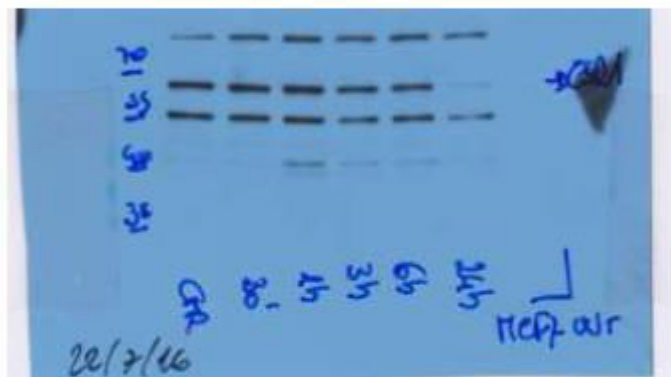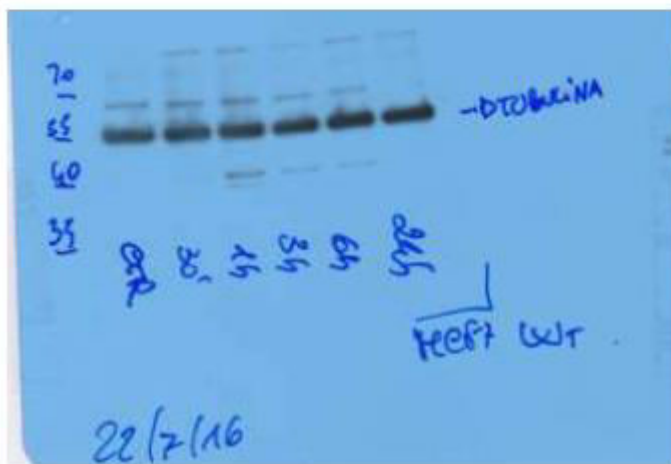

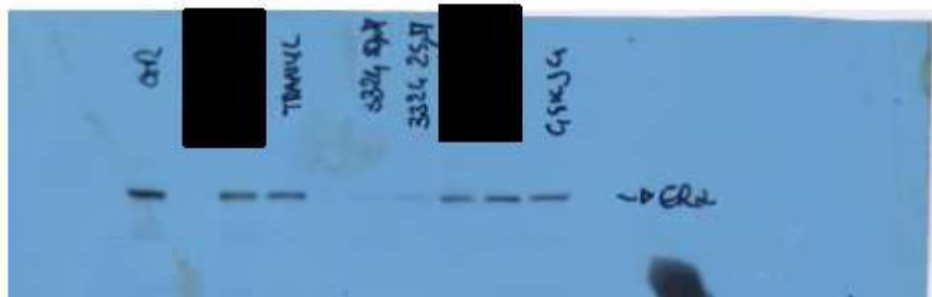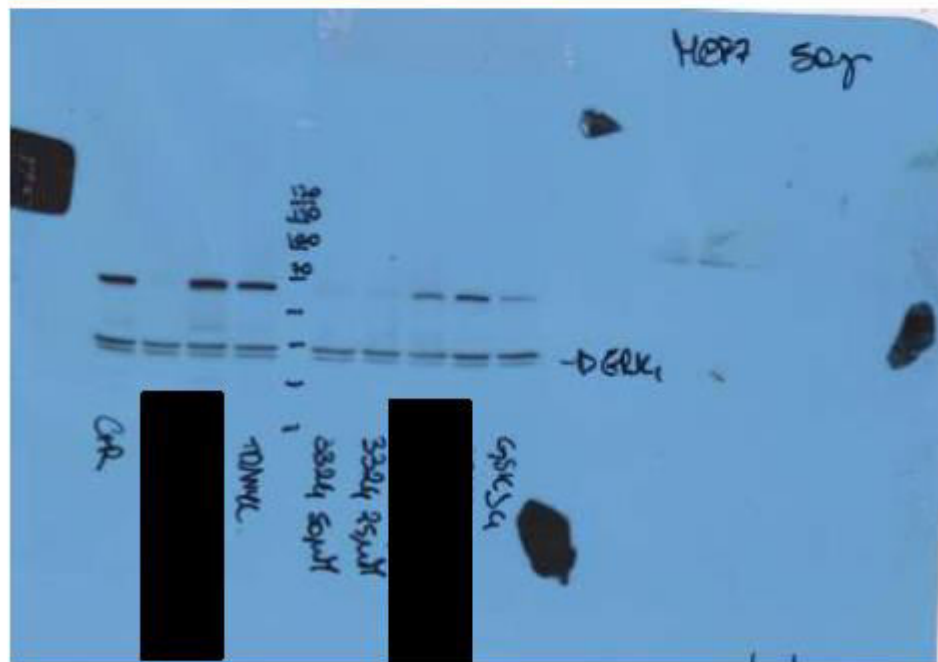

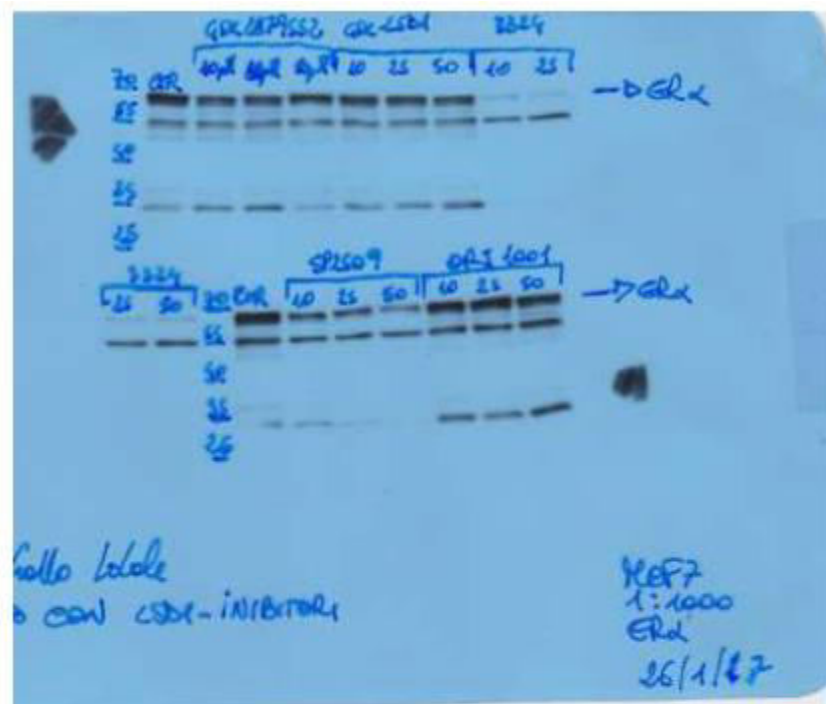

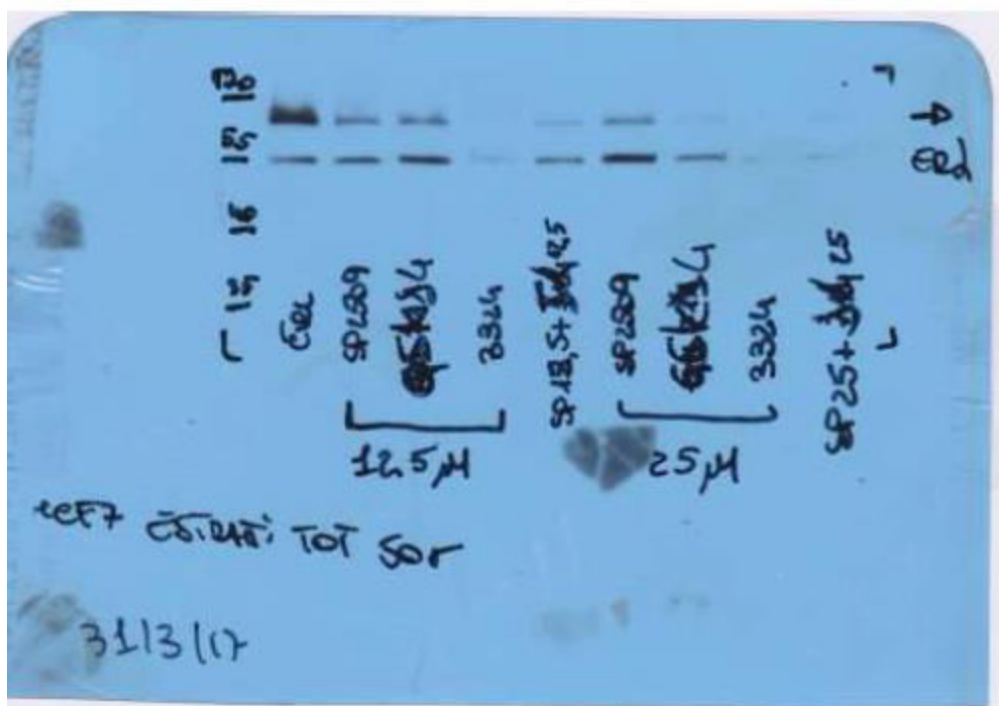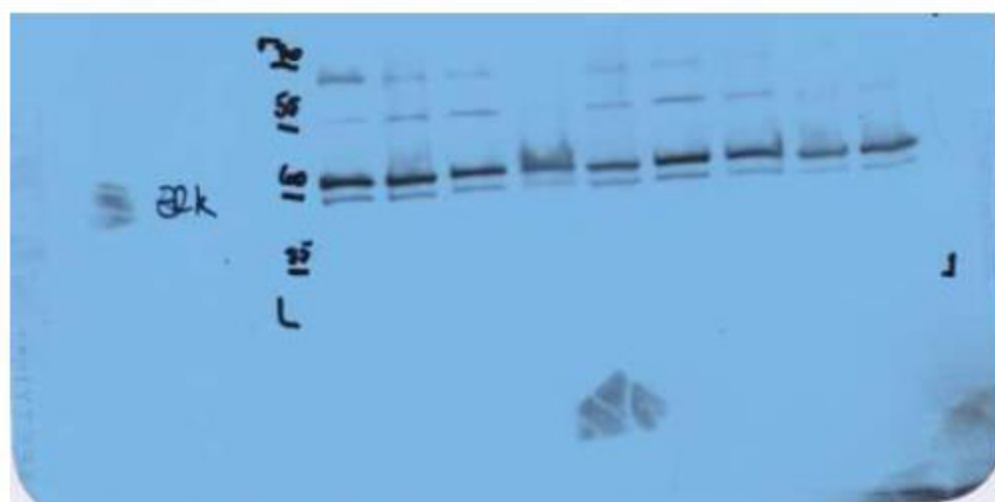

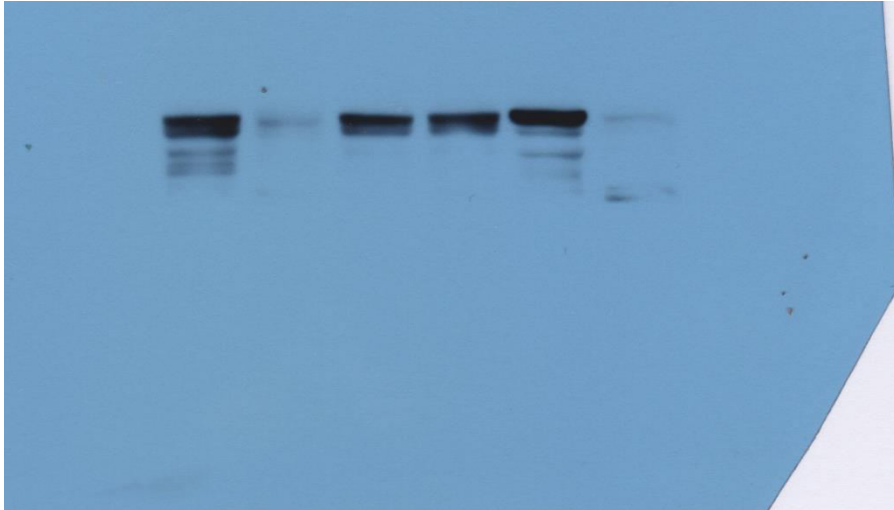

ER $\alpha$

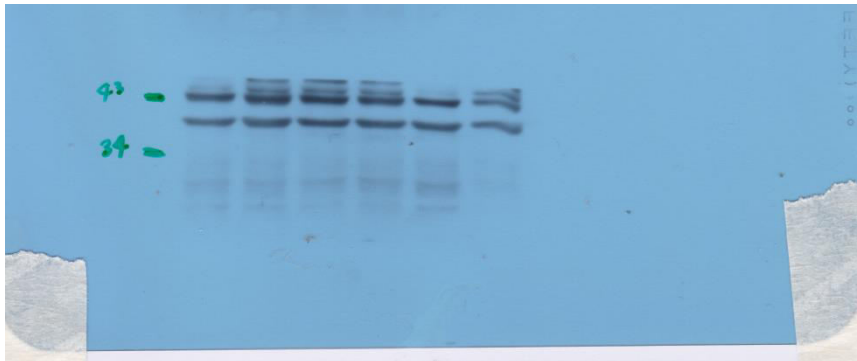

GAPDH

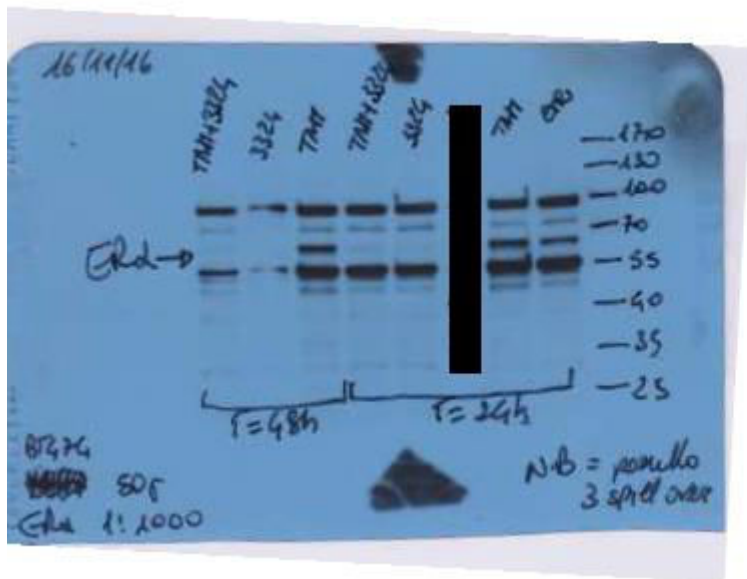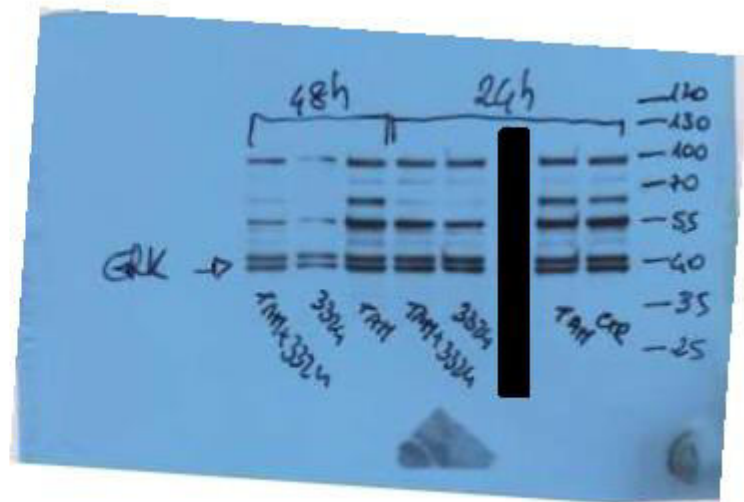

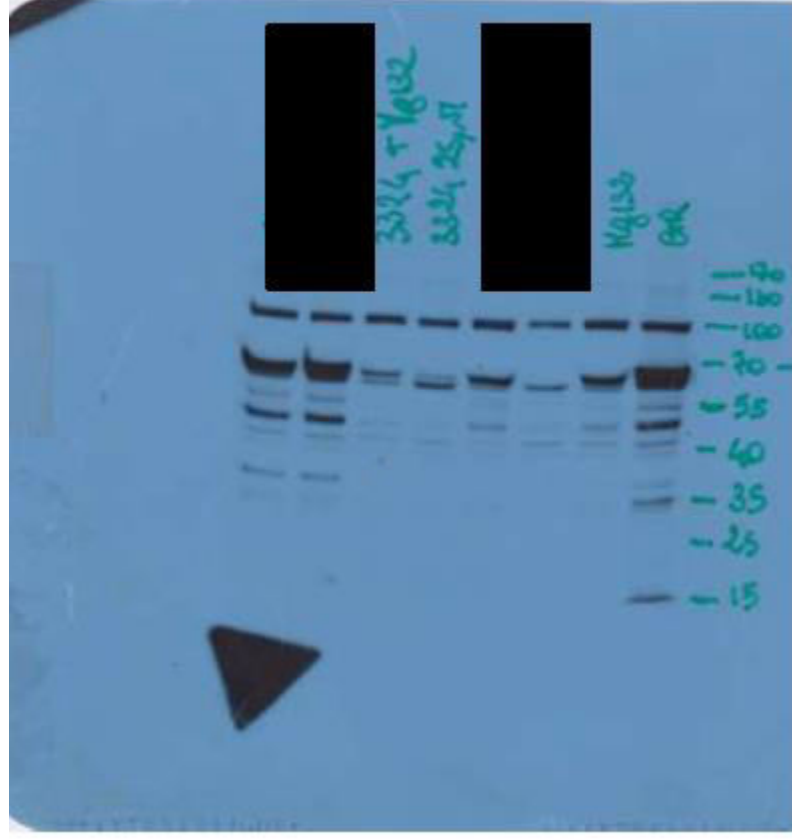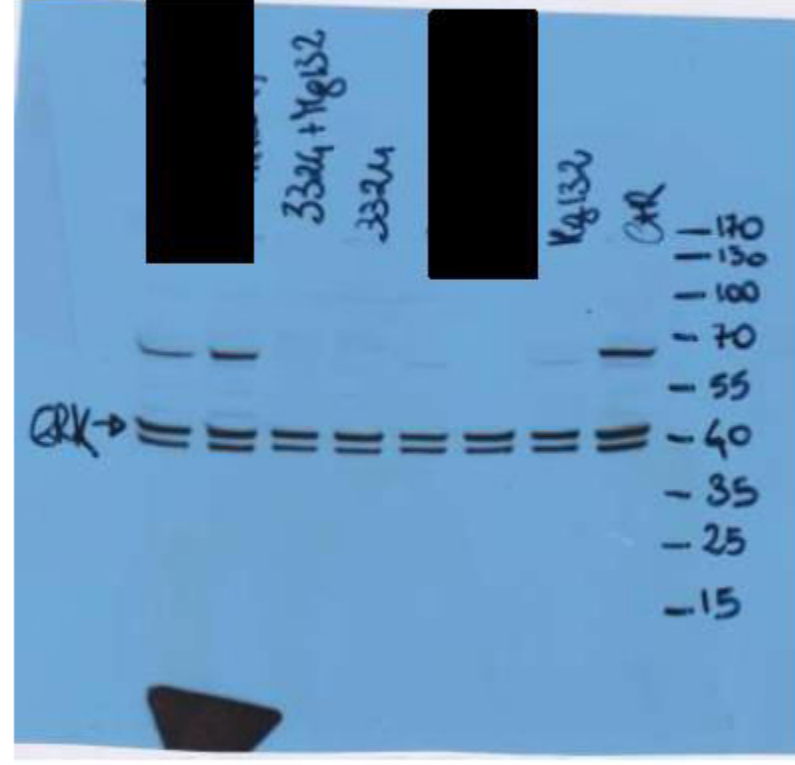

Ab: virginare 1:1000

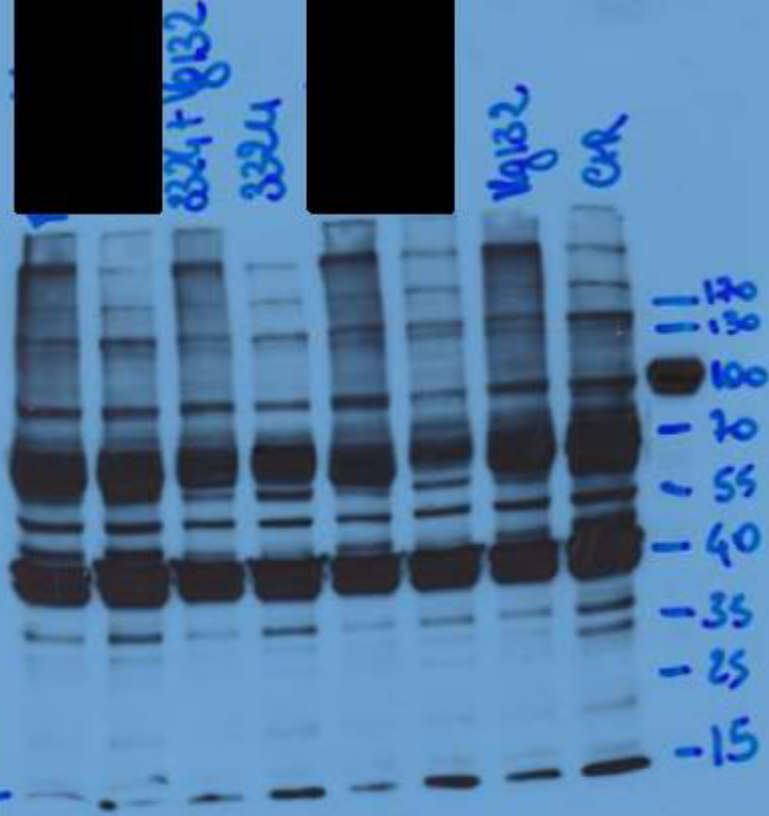

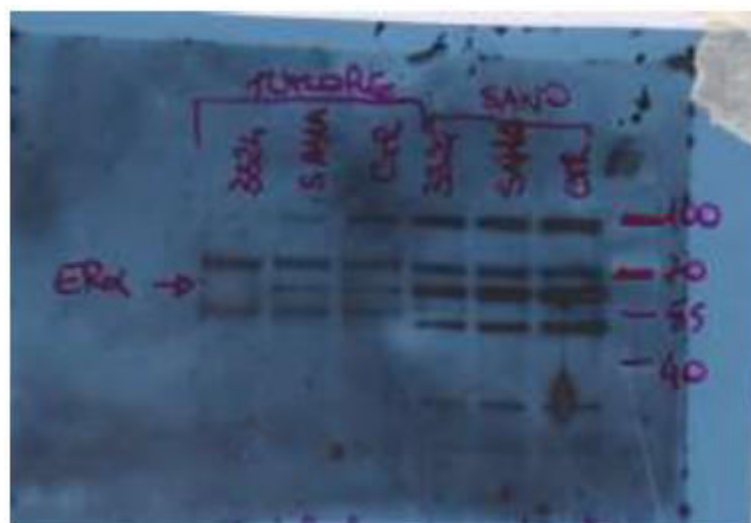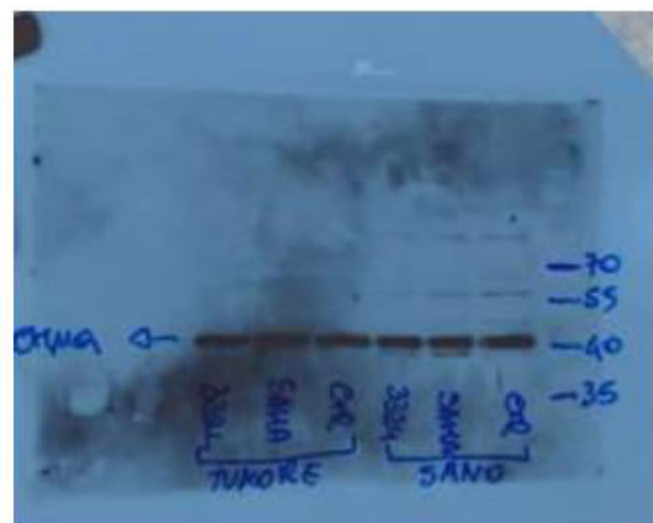

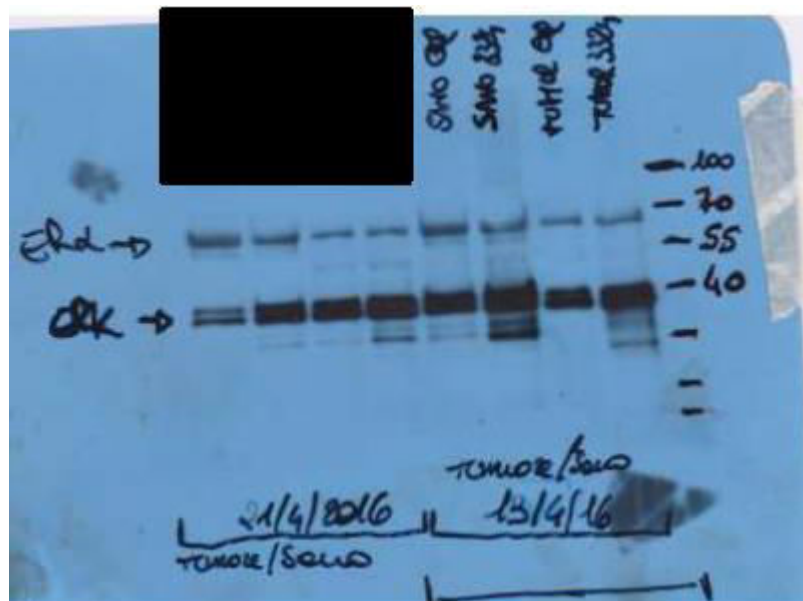

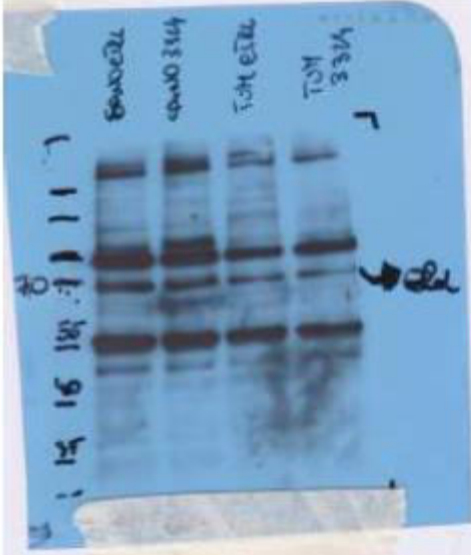

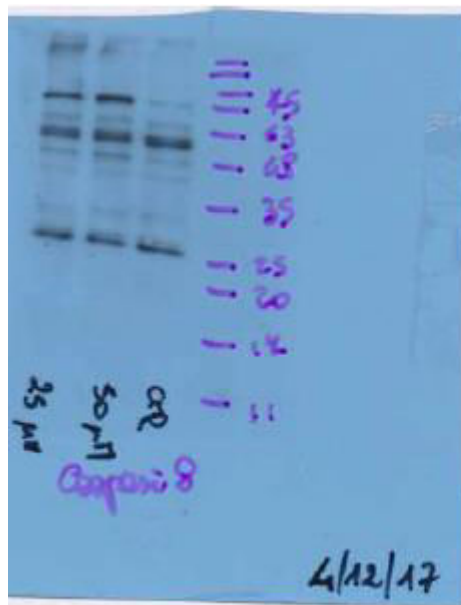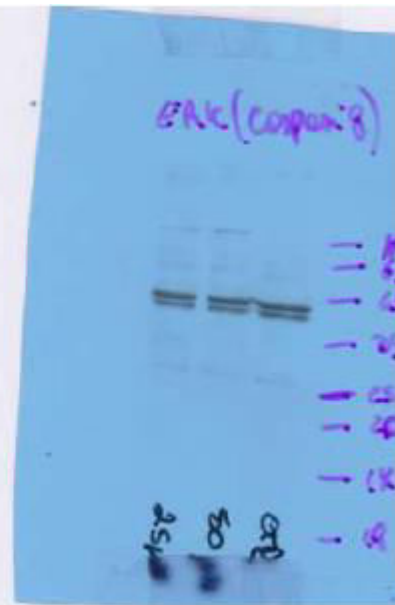

4/12/17

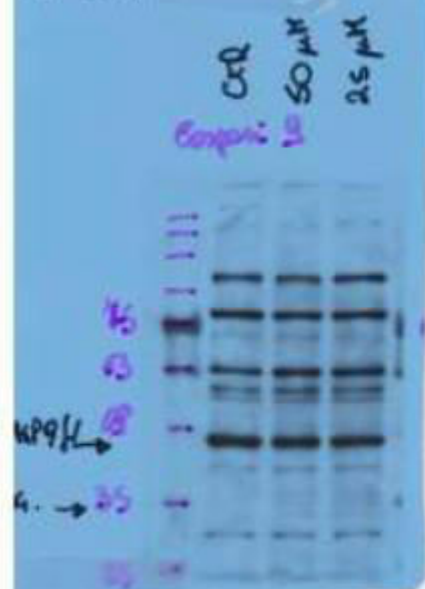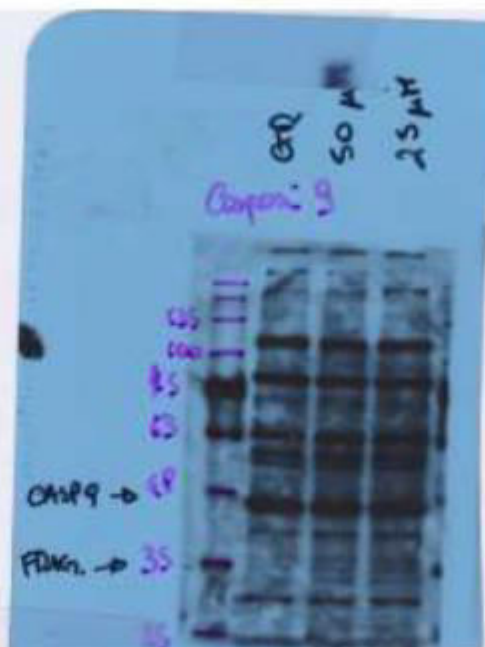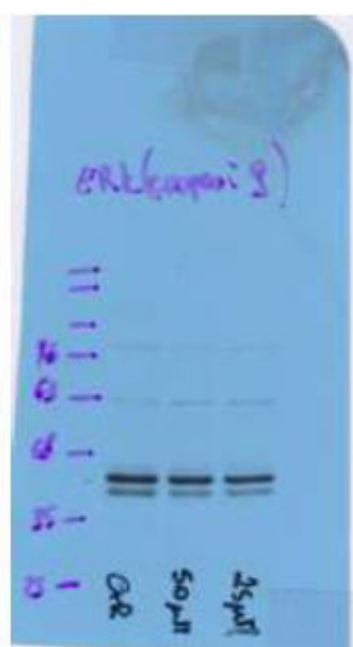

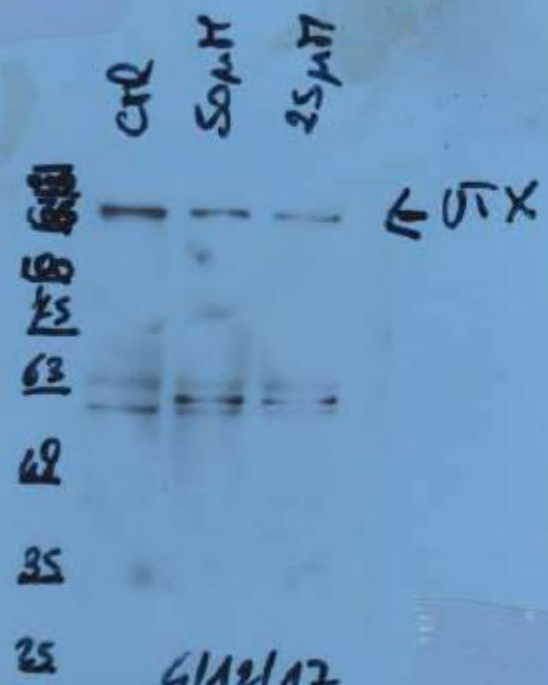

4/12/12

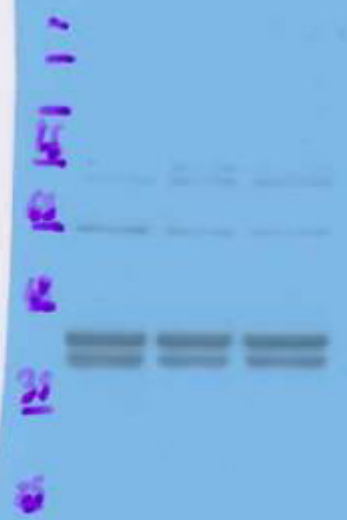

ERK1/2: UTX

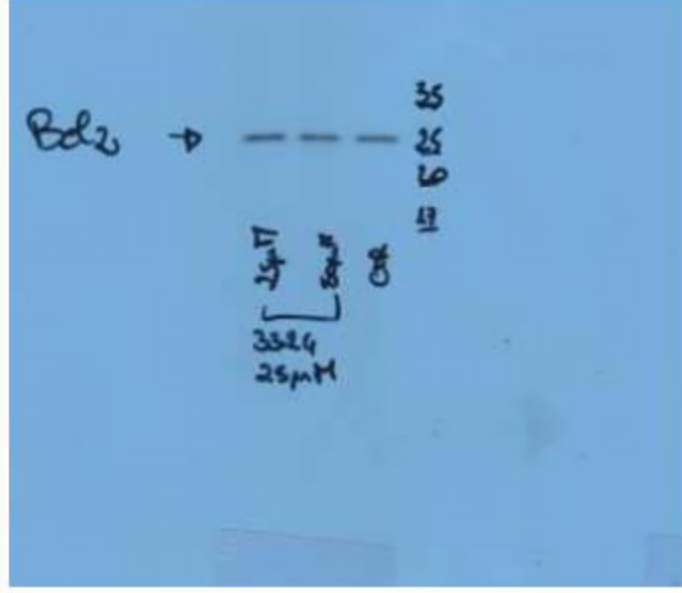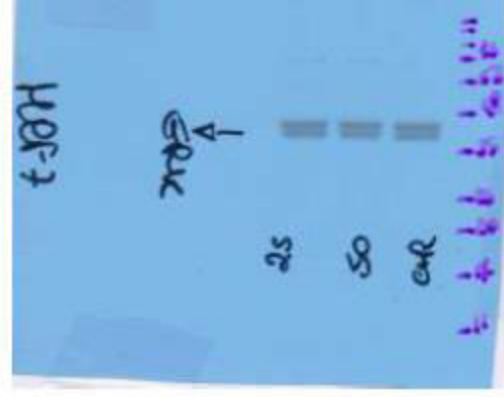

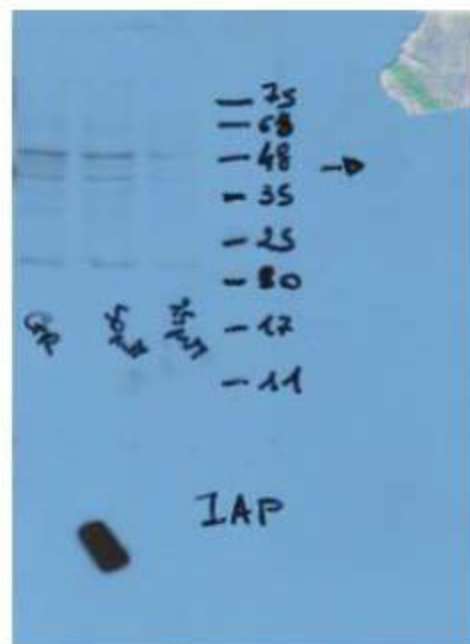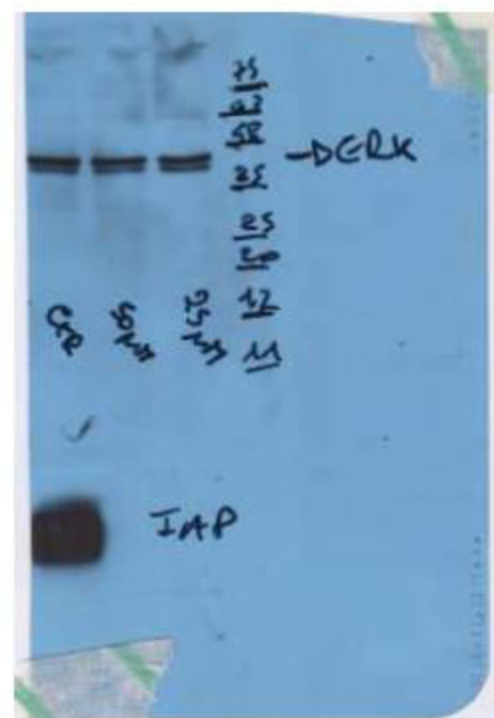

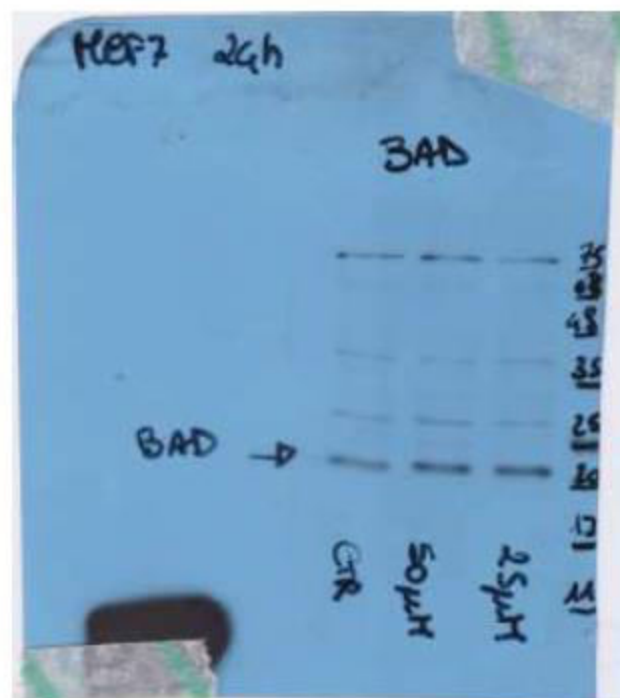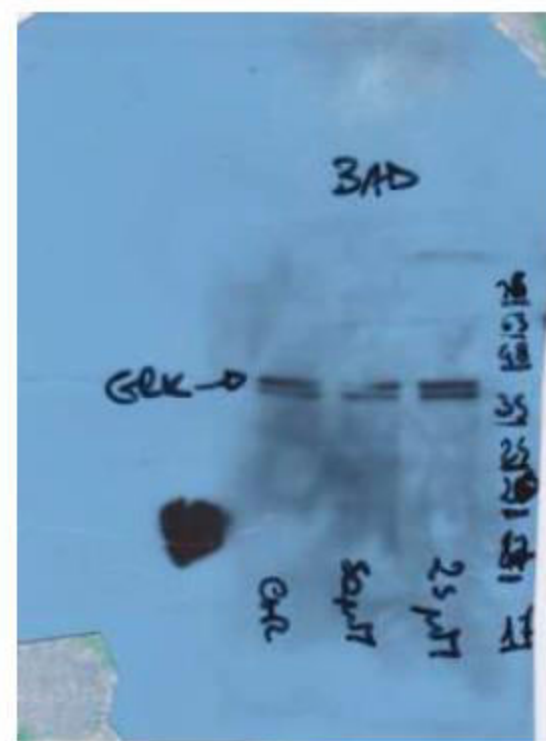

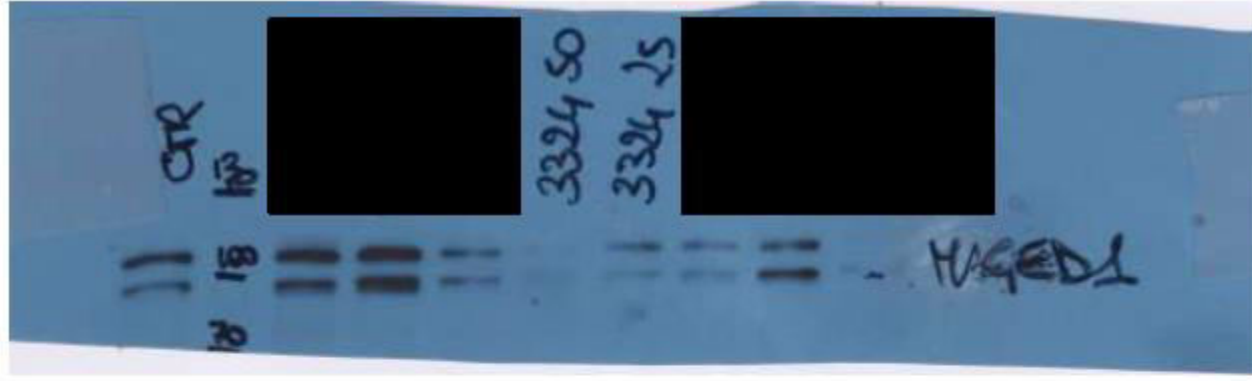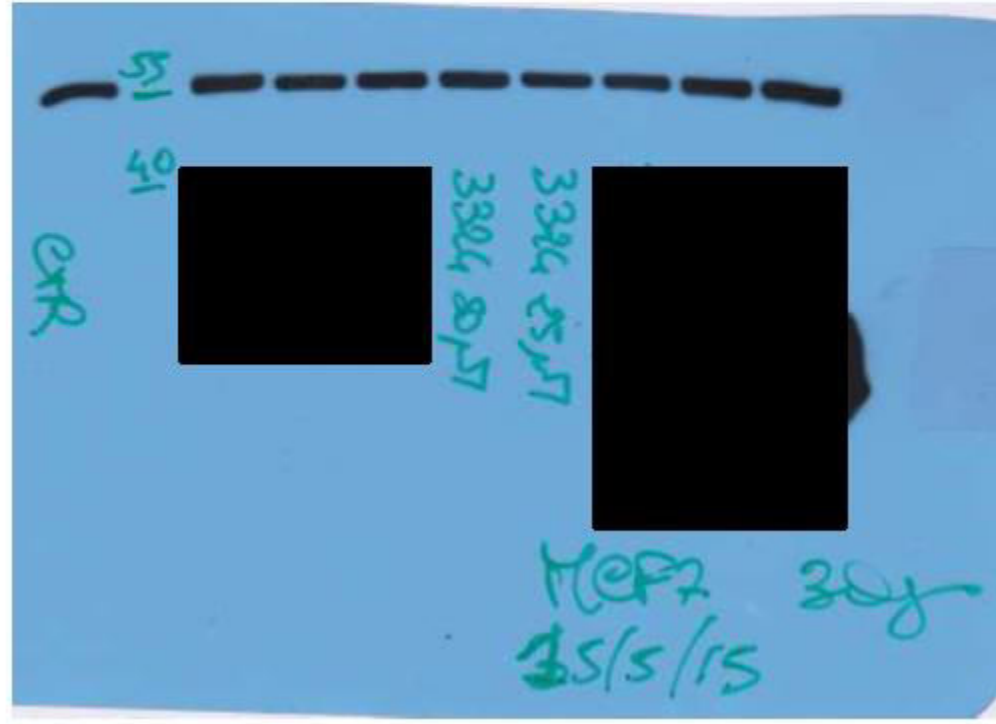



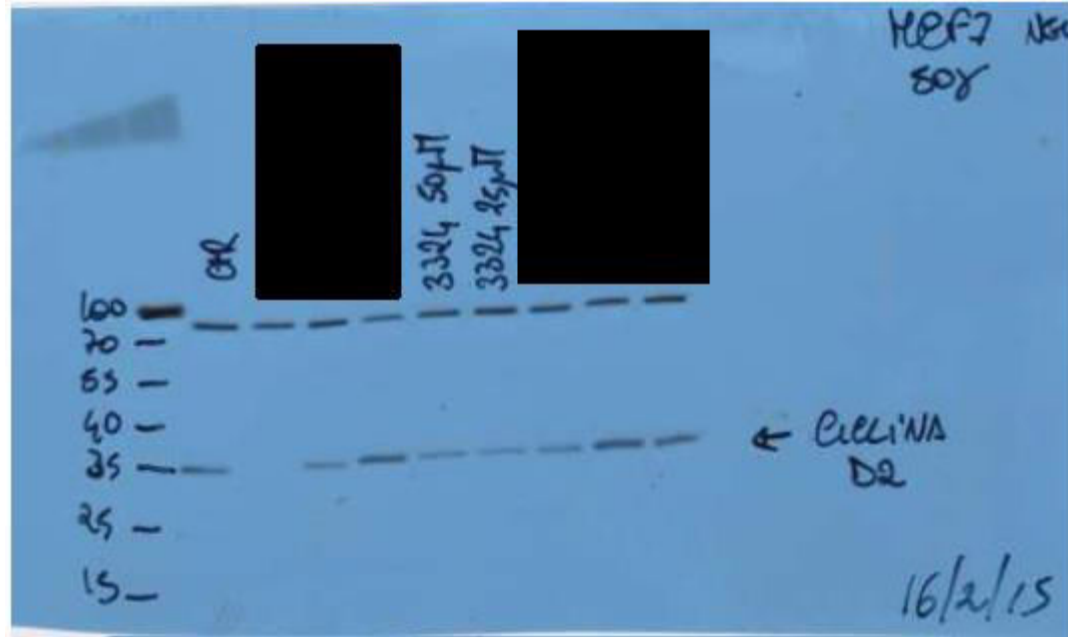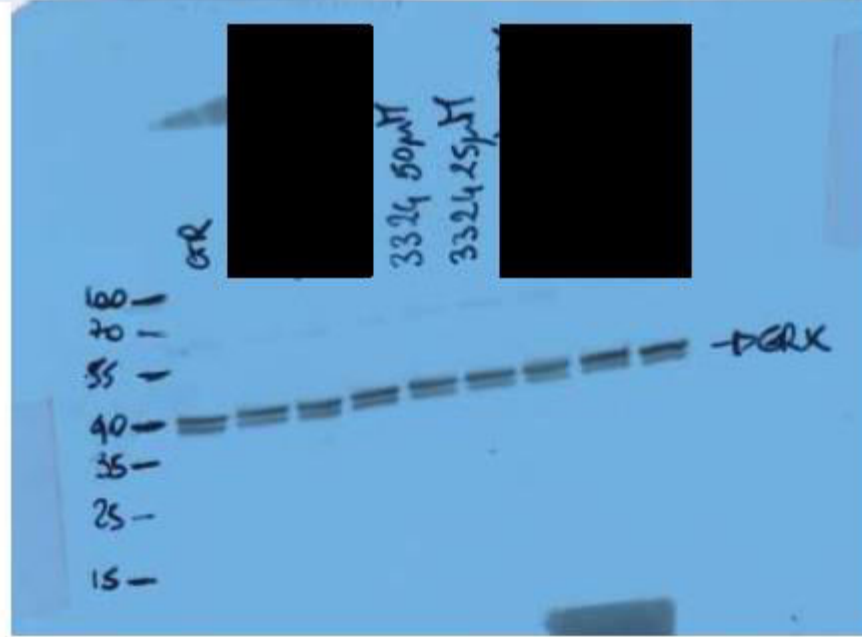

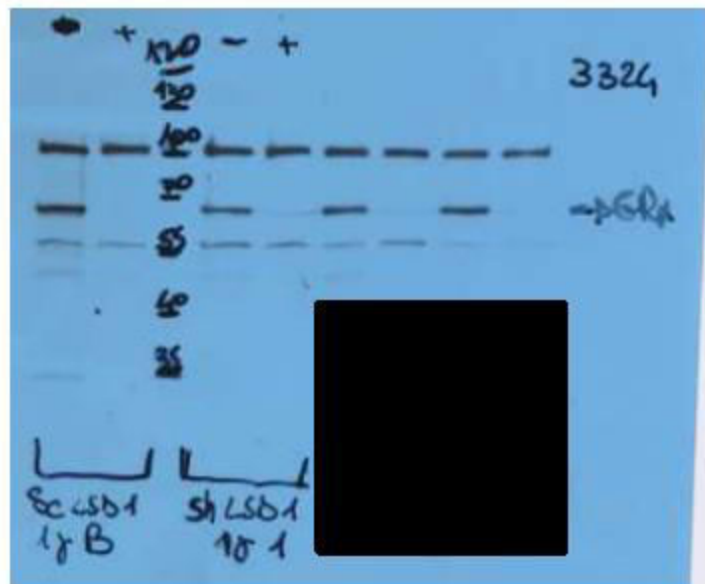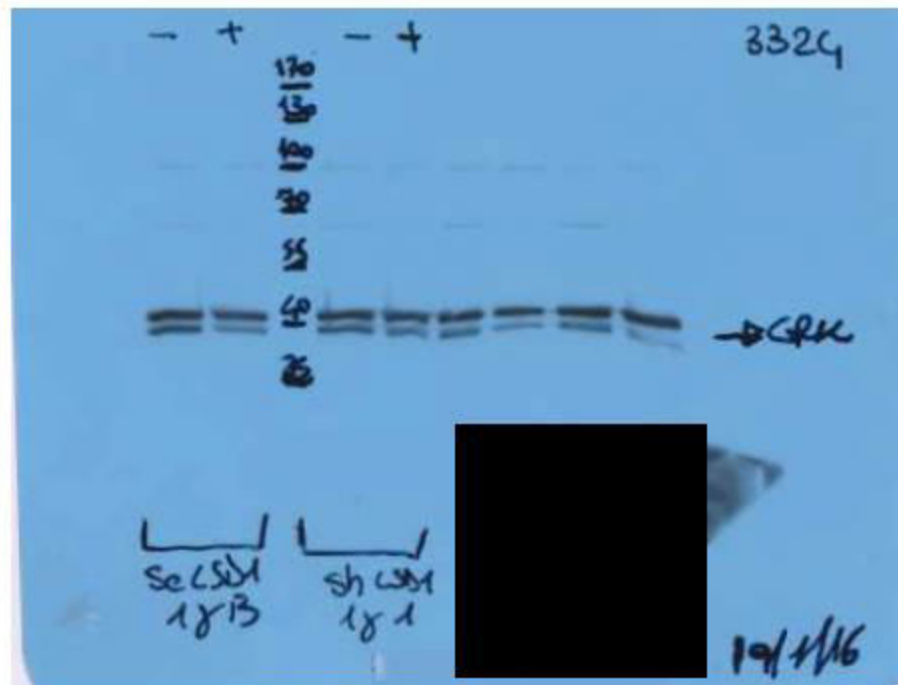

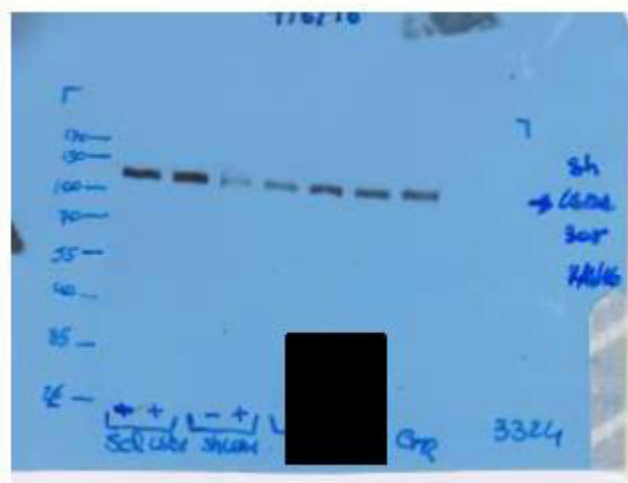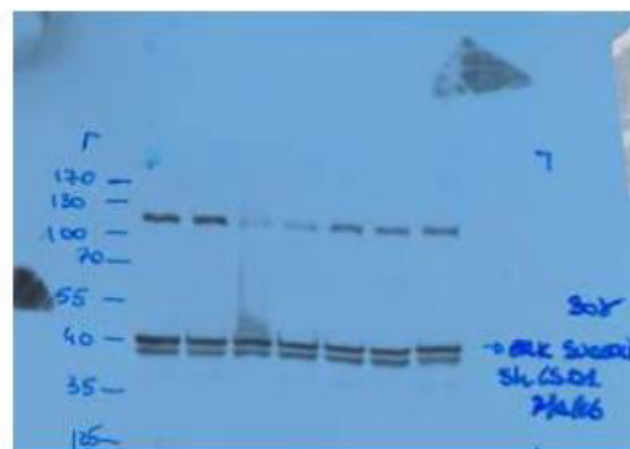

Supplement: Supplementary file 1 [file cancers-11-02027-s001.zip › cancers-659480-supplementary/Original Blots Cancers Compressed.pdf]
